# The role of shape and interaction directionality in the crystalline phase behaviour of octahedral metal-organic cages

**DOI:** 10.1038/s41467-026-77857-7

**Published:** 2026-09-22

**Authors:** Emma H. Wolpert, Andrew Tarzia, Yuhang Zhu, Kim E. Jelfs

**Affiliations:** 1 Department of Chemistry, University College London, London, UK; 2 Department of Materials and the Thomas Young Centre for Theory and Simulation of Materials, Imperial College London, London, UK; 3 I-X Centre for AI in Science, Imperial College London, London, UK; 4 Department of Applied Science and Technology, Politecnico di Torino, Torino, Italy; 5 School of Chemistry, University of Birmingham, Birmingham, UK; 6 Department of Chemistry, Imperial College London, Molecular Sciences Research Hub, White City Campus, London, UK

**Keywords:** Crystal engineering, Computational chemistry

## Abstract

Despite advances in crystal engineering, predicting the packing of metal-containing molecules remains a significant challenge due to their increased structural complexity and computational cost relative to purely organic compounds. Yet, predicting solid-state structures is critical for understanding structureproperty relationships, particularly in modular porous systems such as metal–organic cages (MOCs), where packing governs access to the internal pores and thereby impacts functionality. Here, we present a computationally inexpensive methodology that combines semi-empirical dimer calculations with coarse-grained modelling to predict the packing behaviour of MOCs. This approach enables a priori prediction of the solid state and allows insight into how molecular shape and intermolecular interactions influence the crystalline phase behaviour of MOCs. Our findings provide a foundation for the rational design of MOCs with tailored solid-state structures and properties.

## Abstract (web summary)

The authors combine molecular-dimer calculations with simplified, coarse-grained simulations to predict the crystal structure of metal-organic cages and show how cage shape and the directionality of interactions influence the structures formed.

## Introduction

**Fig. 1:**
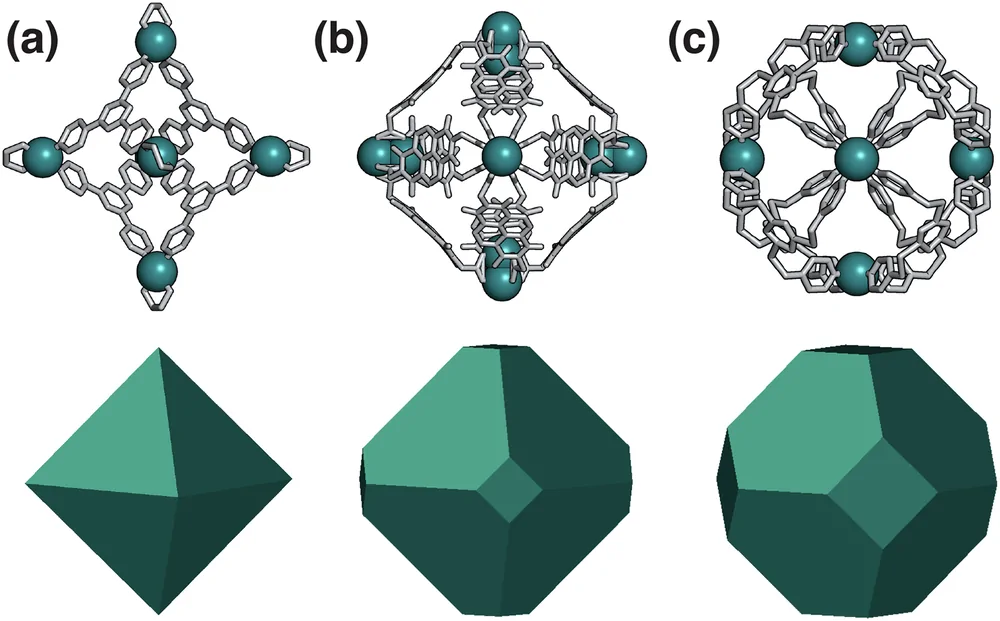
Examples of octahedral MOCs with various levels of truncation and their related truncated octahedra. **a** An example of an M_6_L_4_ octahedral cage^55^. **b** The truncated octahedral MOC [Rh_12_(NDI-Gly)_12_]^24^ and (**c**) a M_6_L_8_-type cage which is related to a more truncated octahedra^39^. Metal ions in each of the cages are shown in teal, all bonds are shown as grey, and hydrogen atoms are omitted for clarity. Teal polyhedra below the cages are their corresponding truncated-octahedron representations.

MOCs are discrete hybrid molecules that are constructed from both metal and organic components and contain an intrinsic void (Fig. 1). This porosity gives them applications in encapsulation^1^, sensing, and separations^2^ while offering receptor sites to aid catalysis^3,4^, enzyme mimicry^5^, and drug delivery^6^. Like other hybrid materials, such as metal–organic frameworks, MOCs offer an enticing playground for both structure and property tuning, thanks to the enormous chemical space of building blocks that can be drawn upon to form them. These discrete cages form via the self-assembly of metal ions and organic ligands into pseudo-polyhedral architectures, typically denoted M_*a*_L_*b*_, where *a* and *b* refer to the number of metal centres (M) and ligands (L), respectively. The geometry of the cage is primarily governed by the coordination preferences of the metal ions and the geometry of the ligands, but subtle changes in ligand chemistry or experimental conditions can lead to the formation of different MOC structures^7^. While predicting the outcome of MOC molecular assembly is a complex process, software and procedures have been developed to help accelerate this process^8–12^. Yet predicting how MOCs crystallise in the solid state remains a major barrier to computational screening, as no standard method currently exists to do so^13^.

Crystal structure prediction (CSP) is often used to predict the solid-state structure of molecular materials, and has successfully predicted the packing of porous organic cages (POCs), the non-metal counterparts to MOCs^14,15^. However, the introduction of metals into the molecules increases the computational expense and difficulty of CSP calculations^13^. Unlike for purely organic compounds, robust and general-purpose atomistic force fields are often not well parameterised for metal-containing systems, especially when it comes to capturing non-covalent interactions and variable coordination environments. Even when parameters exist^16,17^, they can generate hypothetical structures but often struggle to capture the relative stability of isomers, especially those involving transition metals, and can subsequently be unreliable for predicting crystal structures^18^. Calculations therefore require additional cost and expertise, or higher levels of theory such as semi-empirical or density functional theory methods. This can become prohibitively expensive, as MOCs typically contain well over 200 atoms, while CSP studies, even those employing parameterised organic force fields, have historically focused on molecules with fewer than 100 atoms^19,20^.

The challenge is amplified for charged MOCs, where the charge of the metal centres is not fully balanced by charges on the ligands, and a counterion is required in the crystal structure to maintain overall charge neutrality. The inclusion of counterions increases the number of atoms in the asymmetric unit and, consequently, the computational cost. Moreover, reliable energetics often require careful consideration of quantum behaviour, such as spin, which requires (i) an increase in the computational expense and (ii) an increase in the number of calculations to determine the spin state with the lowest energy. Therefore, developing a method to predict the packing behaviour of MOCs without the extensive calculations required by traditional CSP approaches would significantly reduce computational expense, enabling high-throughput screening and accelerating the discovery of MOCs with functional properties in the solid state.

Recently, we developed an approach to predict POC packing that is much less computationally expensive than traditional CSP methods^21^. This methodology coarse-grains the cage structures, representing each cage as a hard polyhedron based on the geometry of the cage, with directional interactions capturing the driving forces behind the solid-state phase behaviour. We later demonstrated that this approach can predict the packing of cages a priori by using atomistic calculations on molecular dimers^22^. These dimer calculations investigate the energetics of different packing motifs between cages and identify the key interactions to be included within the coarse-grained model. Dimer calculations have been used to identify dominant packing motifs in POCs^21–23^, and to parametrise force-field models using results from density functional theory (DFT) calculations^19^. Building on this, our approach incorporates results from force-field, semi-empirical, or DFT-level dimer calculations to inform the coarse-grained model in a dimer-informed coarse-grained modelling protocol. While our previous studies have shown that this approach works well for POCs^21,22^, its application to predicting the packing of metal-containing molecules has not been tested before. Here, we extend our method to MOCs and demonstrate CSP for a metal-containing cage, thus broadening the applicability of our approach to a wider class of materials.

An interesting feature of MOCs is that even though the cage can often be geometrically described as a Platonic solid (e.g., a tetrahedron, octahedron, or cube), the metal-containing nodes can cap the vertices, leading to truncation of the polyhedron (Fig. 1). This capping feature is exemplified by the Rh(II)-based octahedral MOC series [Rh_12_(NDI-X)_12_], reported by Tokuda and Furukawa, where NDI-X is a naphthalenediimide-based carboxylate^24^. In this series, the Rh(II) groups truncate the vertices of the pseudo-octahedral cage (Fig. 1(b)). A key advantage of coarse-grained models is their level of abstraction, which allows model parameters to be tuned continuously. This enables investigation into how changes in molecular chemistry, whether through shape or intermolecular interactions, affect solid-state phase behaviour. For the effect of interactions in cages, we have previously used these models to vary the directionality of the interactions between the cages, and demonstrated that this approach is a useful tool for understanding the polymorphic landscape spanned by POCs^21^. We have not previously explored the effect of geometry, but our coarse-grained model explicitly captures cage shape, enabling us to investigate how features such as vertex capping—and thus truncation—impact solid-state phase behaviour in MOCs.

**Fig. 2:**
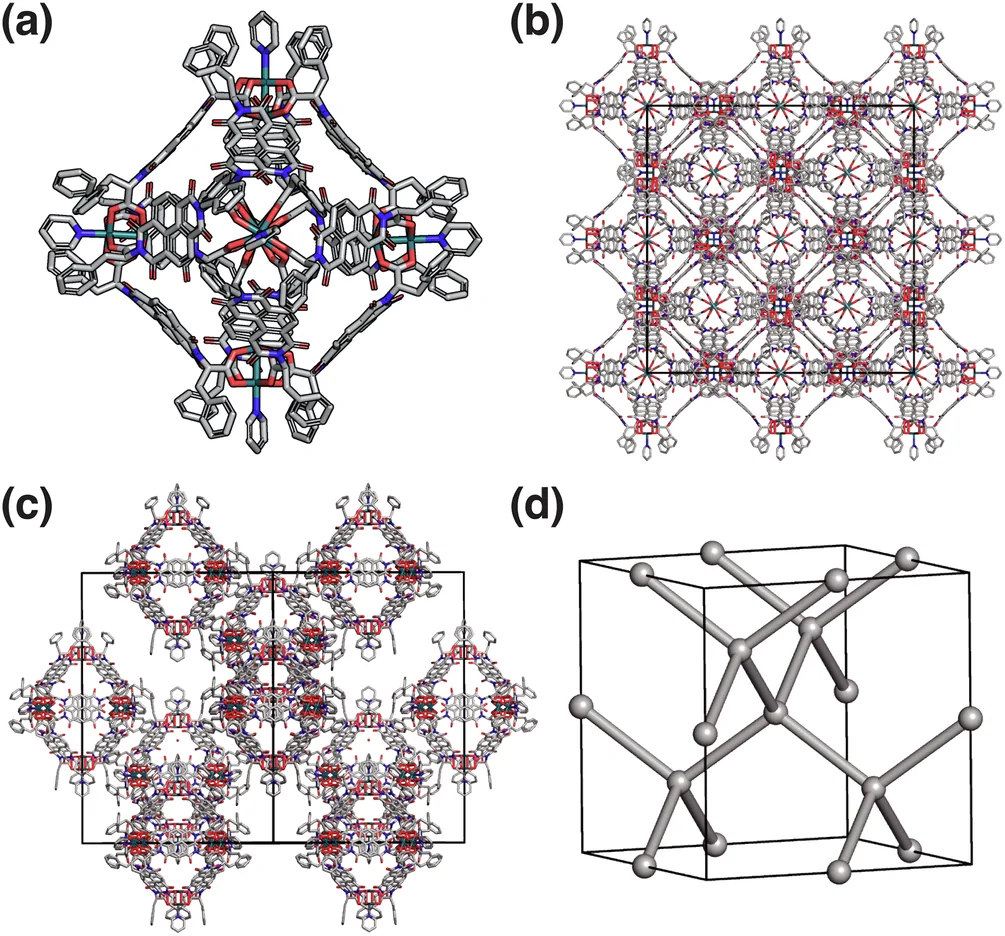
Crystal structure formed by [Rh_12_(NDI-Phe)_12_(pyridine)_6_]. **a** A single molecule of [Rh_12_(NDI-Phe)_12_(pyridine)_6_] and its reported crystal structure, WaaF-1, viewed down the (**b**) [001] and (**c**) [110] directions. This structure crystallises in the *F*4_1_32 space group, forming a diamondoid network between the cages. Bonds in the crystal structures are shown as grey, red, blue, and teal for carbon, oxygen, nitrogen, and rhodium, respectively, with hydrogen atoms omitted for clarity. The network formed by the MOCs is shown in (**d**), where grey spheres represent the centroid of each MOC.

In this study, we focus on the MOC [Rh_12_(NDI-Phe)_12_(pyridine)_6_] as a proof-of-concept case for developing a workflow to predict the packing of MOCs (Fig. 2(a)). Depending on the solvent ratio used in synthesis, this MOC forms two phases: an open framework WaaF-1 (Fig. 2) (named due to its van der Waals open framework), at a 2:1 ratio of ethanol:dimethylacetamide (DMA), and a distorted face-centred cubic (FCC) phase at lower ratios of ethanol:DMA, which collapses into an amorphous solid on evaporation of the solvent^24^. We were particularly interested in predicting the solvent-dependent formation of the WaaF-1 crystal structure, which forms a diamondoid crystal structure in the *F*4_1_32 space group with high extrinsic porosity (Fig. 2(b–d))^24^. This MOC is an especially good candidate for testing our methodology and assessing our ability to predict solvent effects, as it is a neutral MOC with no counterions that retains its diamondoid crystal structure upon desolvation (Fig. 2). The absence of counterions simplifies our dimer calculations due to the reduced number of degrees of freedom. While it is possible to include counterions explicitly, our dimer calculations involve sampling the energies of many cage—cage configurations, and introducing counterions would substantially expand the configurational space by requiring additional sampling over the possible positions of the counterions within each dimer.

Our aims are threefold: (1) demonstrate that the dimer-informed coarse-grained protocol developed for POCs can be extended to cages containing metals, (2) test whether an implicit solvent dimer-informed workflow can predict the packing observed experimentally, and (3) map how molecular shape (through truncation) and interaction directionality influence the packing behaviour and resulting crystal structures of MOCs.

## Results and Discussion

### Dimer calculations

**Fig. 3:**
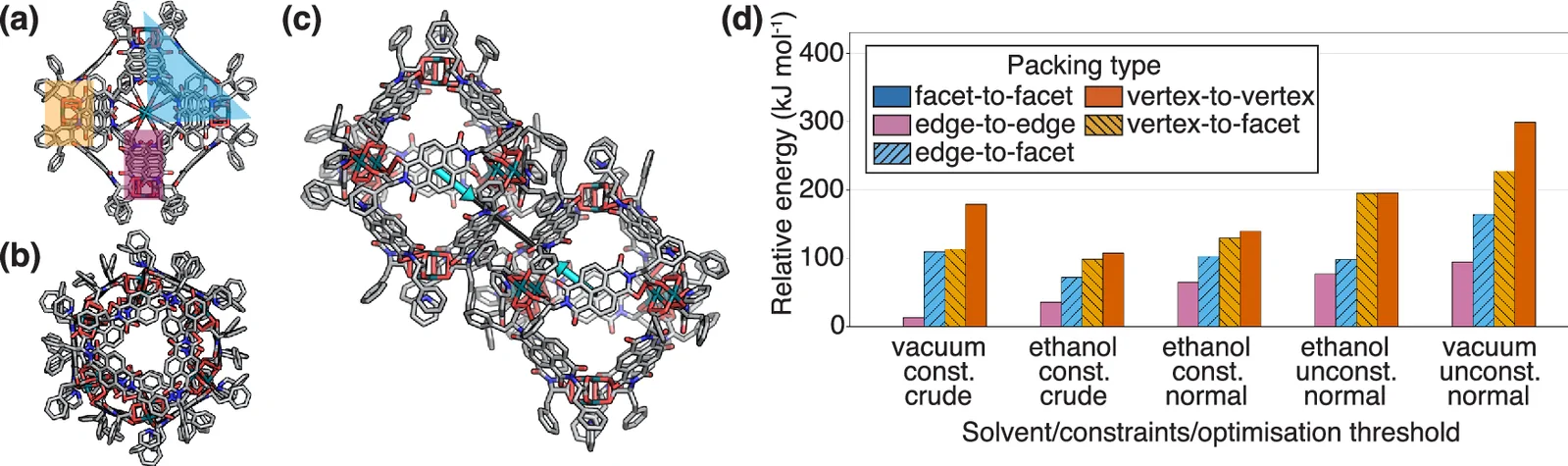
Dimer calculation results. **a** Structure of the MOC [Rh_12_(NDI-Phe)_12_] with one of the facets, edges, and vertices highlighted in blue, purple, and orange, respectively. **b**, **c** Two views of the lowest-energy dimer of [Rh_12_(NDI-Phe)_12_(pyridine)_6_] showing preferred facet-to-facet packing. In (**c**), the cyan arrows show the vector connecting the centroid of each cage to the centroid of the facet. These vectors are well aligned with the vector between the centroids of the two cages, shown in black. Bonds in the molecules are shown as grey, red, blue, and teal for carbon, oxygen, nitrogen, and rhodium, respectively. Hydrogens are omitted for clarity. **d** Energy of the lowest-energy dimer for each packing type relative to the global lowest-energy dimer for each optimisation threshold/constraint choice. const. and unconst. denote constrained and unconstrained metal--metal distances, respectively. The optimisation threshold names come from the xTB software as shown in Table 1. The data used to generate the plot are given in Table S2. Source data are provided as a Source Data file.

Dimer calculations were used to identify the preferred packing motifs of the MOC [Rh_12_(NDI-Phe)_12_(pyridine)_6_] by exploring the configurational space of different cage—cage arrangements around high-symmetry axes: facet-to-facet, facet-to-vertex, edge-to-facet,  edge-to-edge, and vertex-to-vertex (Fig. 3(a) and Fig. S1). For each combination, we examined how the energy varied with the relative rotation of the cages to determine their preferred alignment, and with displacement along the high-symmetry axis to determine their preferred separation, in order to identify the dominant motif. For the high-symmetry facet-to-facet, edge-to-facet, and edge-to-edge combinations, due to the relatively large surface area of the facet/edge, we also explored the energy of configurations which are off-set from the high-symmetry axes. The dimers were created with our open-source code https://github.com/ewolpert1/dimer_calculations, and geometry optimised using the semi-empirical method GFN2-xTB^25^ with constraints on the metal-metal distances within the cages to fix the relative positions of the cages. A full explanation of how we sampled configurational space and details of the calculations are given in the Methods section. Since the packing arrangement of [Rh_12_(NDI-Phe)_12_(pyridine)_6_] is solvent dependent^24^, we incorporated the effect of solvent implicitly by employing the analytical linearised Poisson–Boltzmann (ALPB) model, which adds a solvent-dependent dielectric correction to the electrostatic interactions^26,27^.

The instability of the distorted FCC phase upon desolvation at low ratios of ethanol:DMA suggests that interactions with DMA play a stabilising role, possibly through steric effects as well as weak intermolecular interactions. If steric effects or specific solvent interactions are significant, capturing this behaviour in silico would require an explicit-solvent model of the DMA molecules, which is currently beyond the scope of our method. Therefore, we chose to focus on the effect of ethanol on the packing behaviour as a proof-of-concept test of our methodology for predicting the packing of desolvated MOCs, as the ethanol-solvate crystal structure is stable to desolvation.

Out of the 679 molecular dimers explored, the calculated energies showed that facet-to-facet packing is the most favourable, with the next lowest motif (edge-to-edge) at least 64.8 kJ mol^−1^ higher than the lowest-energy facet-to-facet dimer (with constraints), and thus energetically inaccessible at room temperature (Fig. 3(d)). To assess how directional the facet-to-facet interaction is, we compared the energy and geometry of different low-energy facet-to-facet dimers. Besides the global minimum-energy dimer, several other facet-to-facet arrangements with different off-sets relative to the centroid-facet-facet-centroid axis lie within 65 kJ mol^−1^ (i.e., lower than the edge-to-edge dimer) at 18.9 kJ mol^−1^, 20.1 kJ mol^−1^, and 56.9 kJ mol^−1^ above the lowest-energy dimer. At first glance, this suggests limited directionality, since multiple configurations are low in energy and could be accessible. However, in the lowest-energy constrained dimer, the two cages are not aligned with the centroid axes (Fig. S2). Upon further optimisation, removing the constrained metal-metal distances, the cages align, revealing strong directionality along the axes connecting the facets (Fig. 3(b, c)). In fact, all facet-to-facet structures below the 100 kJ mol^−1^ threshold optimise to approximately the same structure (maximum root-mean-square deviation (RMSD) of 0.156 Å) while edge-to-edge dimers relax to structures at least 77.0 kJ mol^−1^ above the minimum. RMSD calculations were performed excluding H atoms and dynamic groups, e.g., phenyl and pyridine (see the Methods section for further details). The large RMSD between the lowest-energy constrained and unconstrained dimer (0.994 Å) likely explains why a few constrained geometries appeared low in energy, as the constrained minimum was not at the bottom of the true potential-energy surface.

These results indicate that the intermolecular interactions are directional, favouring closely packed, facet-to-facet, antialigned motifs. This is consistent with the analysis of Tokuda and Furukawa^24^, which identified strong van der Waals interactions between the edges of the cage and phenyl groups within the crystal structure, facilitated by the antialignment of the cage facets. The shortest C⋯O intermolecular contact measures 3.17 Å in the experimentally reported structure and the lowest-energy dimer.

We also performed the same optimisation protocol under vacuum to assess the sensitivity of this result to implicit-solvent treatment. These calculations showed the same qualitative energetic ordering as the implicit-ethanol calculations, with the facet-to-facet motif remaining the lowest-energy arrangement. We therefore do not interpret the ALPB calculations as showing that ethanol selectively stabilises the WaaF-1 phase relative to vacuum. Instead, they show that when ethanol is included through the implicit-solvent model, facet-to-facet packing remains preferred, consistent with the experimentally observed packing motif.

### Patchy particle model

**Fig. 4:**
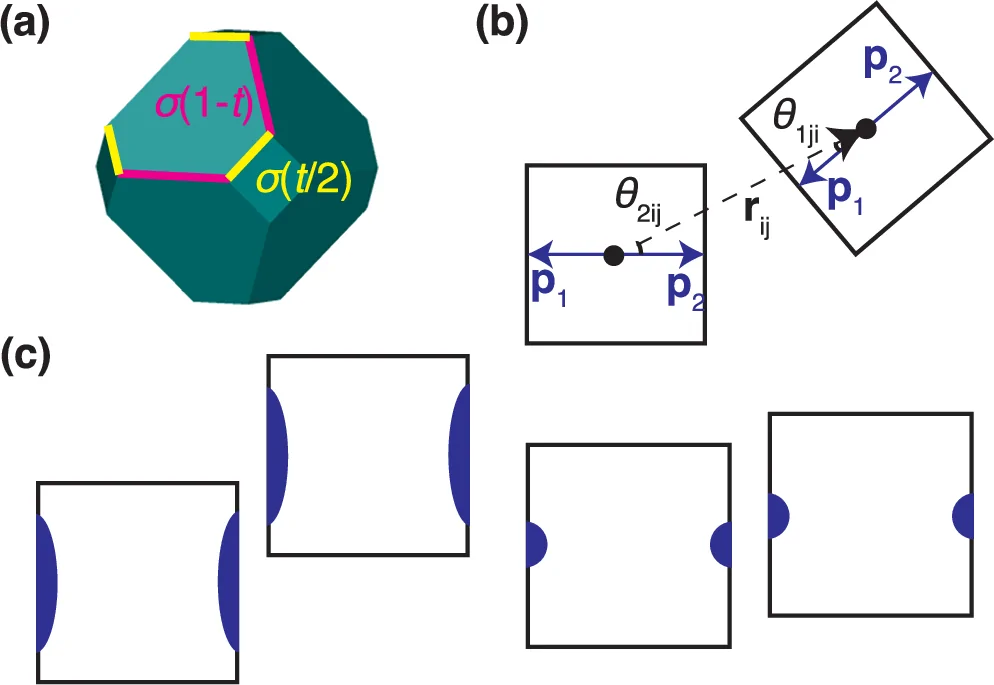
Geometric parameters of the truncated-octahedron patchy-particle model. **a** A truncated octahedron with edge lengths *σ*(1 − *t*) and *σ*(*t*/2), where *t* controls the extent of truncation. **b** Two-dimensional schematic of particles *i* and *j*, each with two attractive patches defined by vectors **p**_1_ and **p**_2_. The centre-to-centre displacement vector **r**_*ij*_ has magnitude *r* = ∣**r**_*ij*_∣. The angles *θ*_2*ij*_ and *θ*_1*ji*_ describe the alignment of the interacting patch vectors on particles *i* and *j*, respectively, with the relevant centre-to-centre direction. These quantities enter the potential defined in equation (1). **c** Illustration of the ranges of relative displacement permitted by large, less directional *σ*_ang_ (left pair) and small, more directional *σ*_ang_ (right pair).

To capture how the facet-to-facet packing observed in our dimer calculations translates to the solid-state phase behaviour of the MOC, we developed a coarse-grained model that represents the cages and simulates their assembly. This model treats each cage as a hard polyhedron with sticky patches, which separates the degrees of freedom of interacting cages into two components: the shape of the cage and the directional interactions responsible for the dominant packing motif. The shape of the polyhedra is defined by a truncation parameter, *t*, where a truncated octahedron has six squares with edge length *σ*(*t*/2) and eight hexagons with edge lengths *σ*(1 − *t*) and *σ*(*t*/2) (Fig. 4(a))^28^. In the simulations, when two polyhedra overlap, the model assigns an infinite potential energy to enforce excluded volume. For non-overlapping configurations, directional interactions are introduced through the patchy particle potential^29–31^. This potential places patches on the facets of the hard polyhedra where the energy is minimised when patches on neighbouring particles point directly at each other. The patches on the hard octahedra were introduced via an interaction term, *V*_*ij*_, which is comprised of the Lennard-Jones potential *V*_LJ_ modulated by an angular term *V*_ang_, and a torsional term *V*_tor_:

1 $${V}_{ij}=\left\{\begin{array}{ll}{V}_{{{{\rm{LJ}}}}}*{V}_{{{{\rm{ang}}}}}*{V}_{{{{\rm{tor}}}}}\quad &r < {r}_{{{{\rm{cut}}}}}\\ 0\quad &r\ge {r}_{{{{\rm{cut}}}}}\end{array}\right.$$

2 $${V}_{{{{\rm{LJ}}}}}=J\left[{\left(\frac{{\sigma }_{{{{\rm{LJ}}}}}}{r}\right)}^{12}-{\left(\frac{{\sigma }_{{{{\rm{LJ}}}}}}{r}\right)}^{6}\right]$$

3 $${V}_{{{{\rm{ang}}}}}=\exp \left(-\frac{{\theta }_{\alpha ij}^{2}}{2{\sigma }_{{{{\rm{ang}}}}}^{2}}\right)\exp \left(-\frac{{\theta }_{\beta ji}^{2}}{2{\sigma }_{{{{\rm{ang}}}}}^{2}}\right)$$

4 $${V}_{{{{\rm{tor}}}}}=\exp \left(-\frac{1}{2{\sigma }_{{{{\rm{tor}}}}}^{2}}{\left[\min ({\phi }_{\alpha \beta }-{\phi }_{\alpha \beta }^{{{{\rm{offset}}}}})\right]}^{2}\right).$$

Here, *V*_ang_ and *V*_tor_ are dimensionless orientational modulation terms rather than independent energetic contributions, such that they switch the Lennard-Jones attraction on or off depending on the relative angular and torsional alignment of the patches. In these equations, *α* and *β* are patches on particles *i* and *j*, respectively (Fig. 4(b)), and the parameter *σ*_LJ_ is the measure of the diameter of the interacting particles without the inclusion of the hard-particle model. Given that the dimer calculations showed a preference for close packing, we set *σ*_LJ_ = 0.5 Å such that the equilibrium bond length is approximately the hard-particle limit of $1/\sqrt{3}$ as *σ*, which defines the edge lengths in Fig. 4(a), was set to 1 Å. The cutoff distance, *r*_cut_, is set to 2.2*σ*_LJ_.

*V*_ang_ and *V*_tor_ modulate the Lennard-Jones potential by reducing the magnitude of the attractive interaction for configurations that misalign the patches (*V*_ang_) or introduce rotations away from the preferred alignment (*V*_tor_). *θ*_*αij*_ is the angle between the vector connecting the two particles' centroids, **r**_*ij*_ with magnitude *r*, and the patch vector (see Fig. 4(b)). ${\phi }_{\alpha \beta }^{{{{\rm{offset}}}}}$ is the preferred torsional angle between patches *α* and *β*, whereas *ϕ*_*αβ*_ is the actual torsional angle between patches *α* and *β*, such that there is an energy minimum where the two angles are the same. The preferred torsional angle in our simulations was *π*/3, as the facets prefer to be anti-aligned.

As both *V*_ang_ and *V*_tor_ are Gaussian functions, *σ*_ang_ and *σ*_tor_ dictate the energetic penalty of particles deviating from being perfectly aligned, as changing *σ*_ang/tor_ narrows or widens the Gaussian function that describes the patchy interaction. This effectively changes the angular/torsional width that the particles interact through, tuning the directionality of interaction (Fig. 4(c)). Through this work, we examine how changing *σ*_ang_ between 0.1−1.0 affects the solid-state phase behaviour of the interacting octahedral particles and set *σ*_tor_ = 2*σ*_ang_ as in our previous studies^21,22^.

Smaller values of *σ*_ang/tor_ narrow the Gaussian function in Eqn. (3), which results in a smaller value of *V*_ang/tor_. Therefore, changing *σ*_ang_, and thus *σ*_tor_, causes clusters to be formed at different temperatures for the same interaction parameter, *J*. The truncation, *t*, can also affect the transition temperature, as the competition between shape/interactions can cause transition temperatures to vary for the same value of *σ*_ang_. Considering the absolute energy scale is not important for our results, for simplicity, we chose to vary *J* for different values of *σ*_ang_ and *t* such that the transition temperature *T*_t_ occurred when *k*_B_*T*_t_ ≈ 1. The interaction parameters used are summarised in Supplementary Table S1.

### Crystal structure phase diagram

**Fig. 5:**
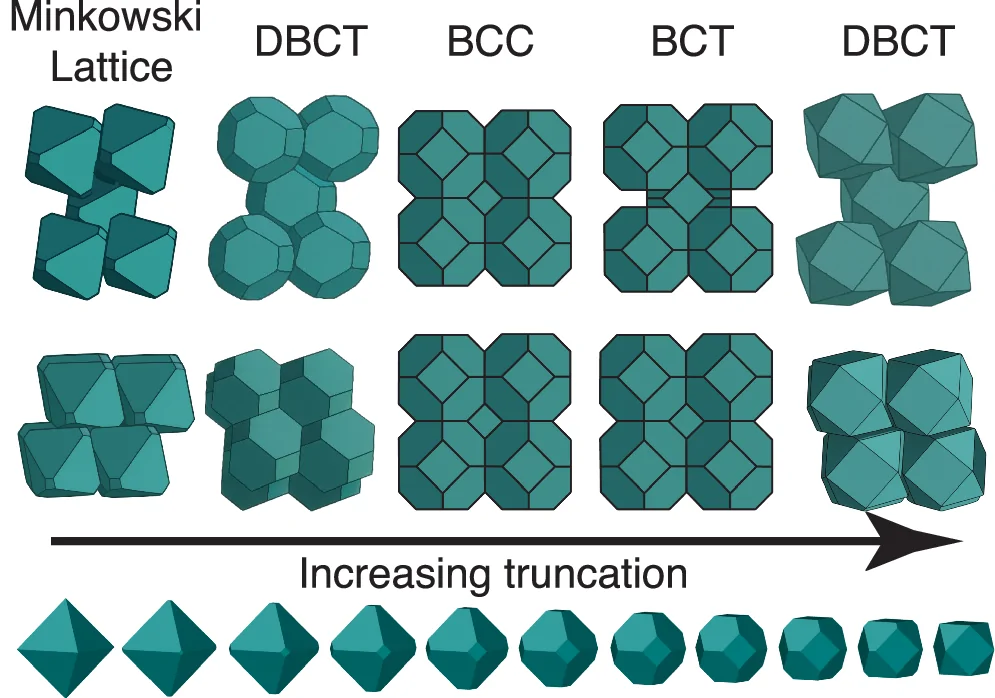
Crystal structures formed by hard truncated octahedra. Above: Graphical depictions of the different phases as reported for high-pressure simulations of hard octahedra as a function of truncation with no interactions by ref. ^34^. Selected elements were adapted and recoloured from Gantapara et al., J. Chem. Phys. 142, 054904 (2015), https://doi.org/10.1063/1.4906753, with permission from AIP Publishing. Copyright 2015 AIP Publishing LLC. From left to right, the Minkowski lattice, disordered body-centred tetragonal (DBCT), body-centred cubic (BCC), body-centred tetragonal (BCT), and DBCT phases. The top and bottom rows are related by a 90^∘^ rotation about the *x*-axis. Below: Octahedron truncated from *t* = 0 (left) to *t* = 1 (right) in 0.1 increments.

To explore how shape and interaction directionality influence the solid-state phase behaviour, we ran multiple simulations varying the level of truncation, *t*, of the hard polyhedra, between *t* = 0−1 (Fig. 5), as well as the directionality of the interactions *σ*_ang_ = 0.1−1.0. In our model, the smaller *σ*_ang_, the more directional the interaction between the facets. Based on the results from the dimer calculations, we expect the phase corresponding to the WaaF-1 crystal structure to emerge at low *σ*_ang_ (high directionality). To estimate the extent of truncation, we measured the distance between the carbon atoms in the carboxylates at each end of an NDI ligand, and the distance between carboxylate carbons of neighbouring NDI ligands coordinated to the same metal centre, corresponding to *σ*(1 − *t*) and *σ*(*t*/2) respectively (Fig. 4(a)). This gave an estimate of *t* ≈ 0.39.

Using these variables, we performed hard particle Monte Carlo (HPMC) simulations in the NVT ensemble with the simulation toolkit HOOMD-Blue^32,33^. The HPMC simulations employ the Metropolis Monte Carlo algorithm, where trial moves are either translations or rotations of the hard particles. Simulations contained 512 particles in cubic boxes with periodic boundary conditions and started from *k*_B_*T* = 1.35 before being gradually cooled through the transition point *k*_B_*T* ≈ 1, where the particles would cluster and a crystal structure would form. The temperature at each step, *T*_*i*_, was reduced according to T_i+1_ = 0.95*T*_*i*_. Further details about the simulations are given in the Methods section.

To contextualise our results, we refer to the work of Gantapara et al.^34^, who systematically studied the phase behaviour of hard octahedra at varying levels of truncation under pressure in the absence of interactions. They identified five distinct phases at high pressure as a function of truncation: the Minkowski lattice—the densest packing of ideal octahedra—occurring for truncation levels between *t* = 0 to *t* = 0.58, a disordered body-centred tetragonal (DBCT) phase between *t* = 0.58−0.66, a body-centred cubic (BCC) phase at exactly *t* = 0.66, a body-centred tetragonal (BCT) phase between *t* = 0.66−0.92, and a second DBCT phase between *t* = 0.92−1 (Fig. 5). We compare our findings to these results to understand how the introduction of directional interactions alters the known phase landscape of hard-particles.

**Fig. 6:**
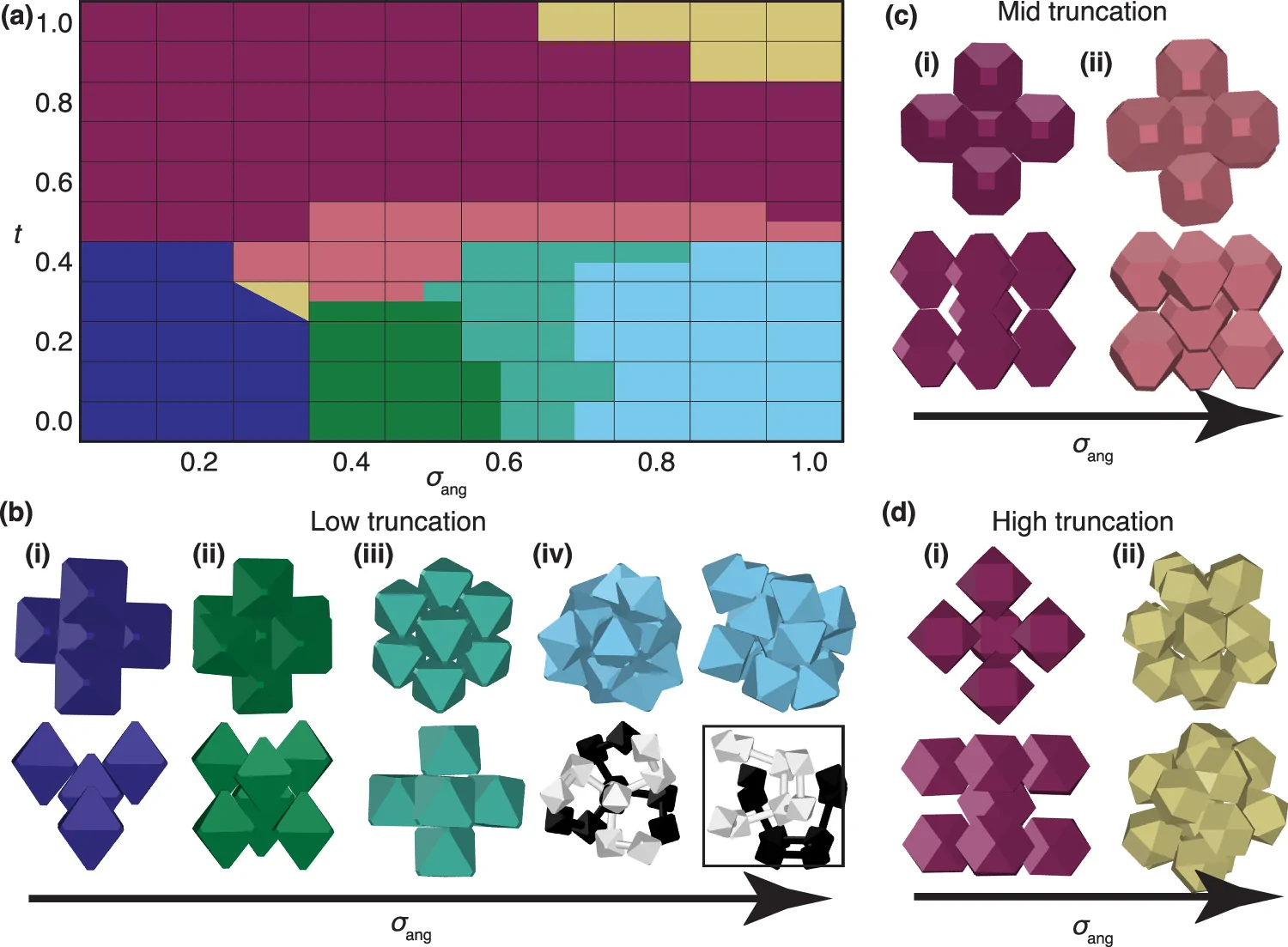
Phase diagram and representative clusters produced from the coarse-grained model. **a** Phase diagram showing the structures formed as a function of truncation (*t*) and *σ*_ang_ (interaction directionality). Each colour represents a distinct structure obtained from simulation. Representative clusters of the different phases for (**b**) low truncation, shown as *t* = 0.2, (**c**) mid truncation, shown as *t* = 0.5, and (**d**) high truncation, shown as *t* = 1.0. Within each truncation regime, subpanels (i–iv) in (**b**), (i–ii) in (**c**), and (i–ii) in (**d**) show the distinct phases encountered with increasing *σ*_ang_ from left to right. The two orientations for each phase are related by 90^∘^ rotations about the *x*-axis, apart from for the light blue phase at high *σ*_ang_ in (**b**). For the light blue phase, the orientations correspond to the view along the [111] (left) and [100] (right) vector in the geometrically equivalent structure formed by lithium under high pressure, as reported by Hanfland et al.^36^. Below, the size of the polyhedra is scaled down to highlight the two sublattices with facet-to-facet interactions in black and white. The black box around the view down the [100] axis is the periodic unit cell of the crystal structure. The colours of the clusters correspond to the different phases as in the phase diagram. Source data are provided as a Source Data file.

For our analysis, we examined the final configuration from each simulation, and clustered these structures using unsupervised machine learning based on their radial distribution functions (RDFs) (see the Methods section for details on how they were clustered). This produced the phase diagram shown in Fig. 6(a). To ensure consistency, each simulation was run five times. For some combinations of *σ*_ang_ and *t* at the boundaries between phases, the same input parameters sometimes led to multiple phases, which we discuss in more detail later. We note that this study does not give a complete overview of the phases formed by all truncated octahedral MOCs, as it focuses on the structural behaviour of polyhedra with directional interactions between the facets and short intermolecular distances.

**Fig. 7:**
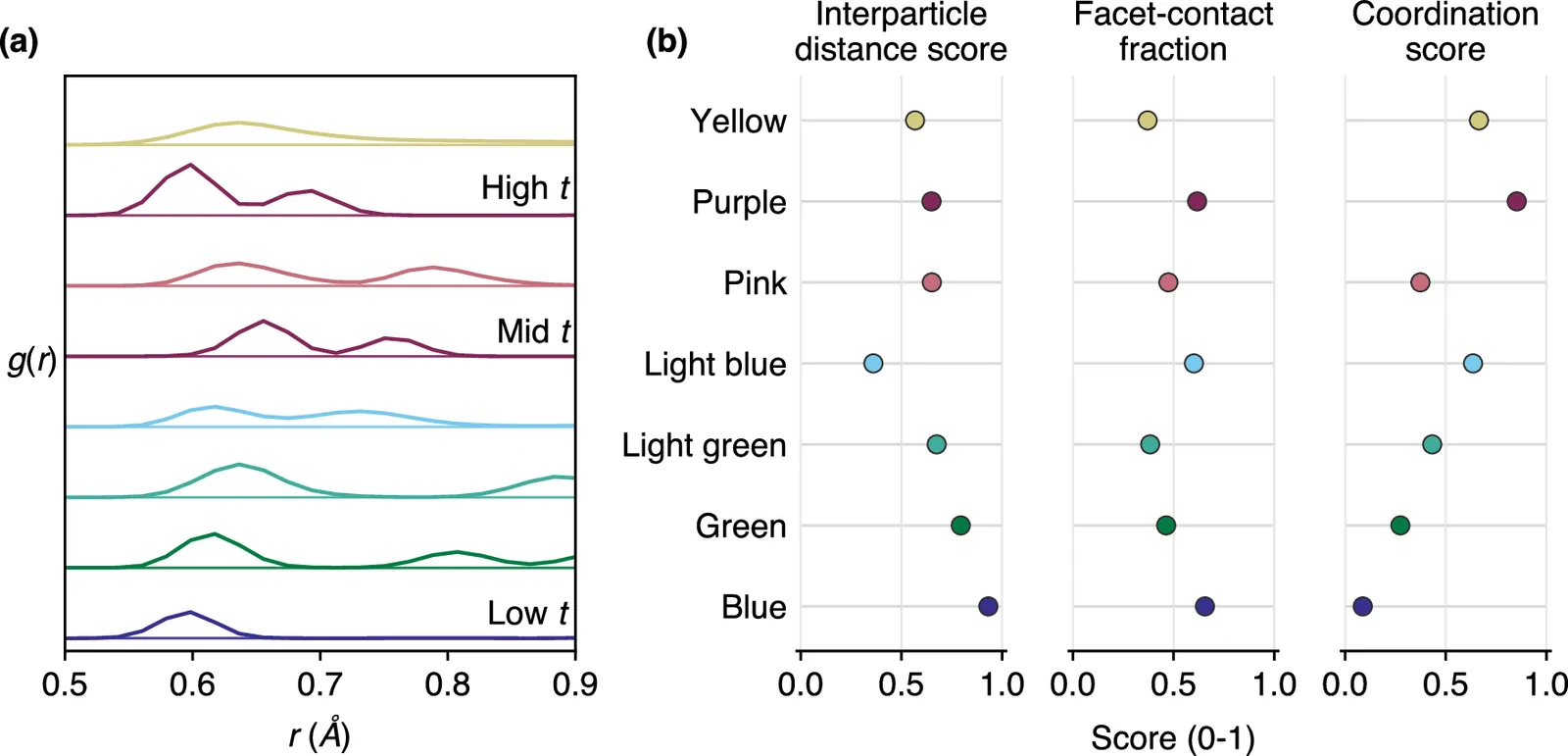
Radial distributions and structural descriptors of the different phases. **a** Representation of radial distribution functions, *g*(*r*), for the nearest-neighbour shells, where *r* is the interparticle distance and *t* is the truncation parameter. **b** Dot plots comparing the structural descriptors—interparticle-distance score, facet-contact fraction and coordination-number score—of each simulated phase. The descriptors are scaled from 0 to 1 across the full dataset, and the interparticle distance is inverted so that larger values represent shorter interparticle distances. Each coloured trace represents the average score for one phase. The colours of the plots correspond to the different phases as in the phase diagram shown in Fig. 6. Source data are provided as a Source Data file.

The seven structures highlighted in our phase diagram each occupy distinct, continuous regions of phase space (Fig. 6(a)). Example clusters of each of the seven phases as formed in the simulations are given in Fig. S3. To analyse the differences between the phases, we examined representative clusters from each simulation, focusing on the coordination number (Fig. 6(b–d)). We also looked at the RDFs of the nearest-neighbour shells (Fig. 7(a)) and the degree of contact between the facets of neighbouring polyhedra expressed as the area of contact divided by the total area of the facet (Fig. S6). Truncation of the octahedra emerged as the dominant factor shaping phase behaviour, with a clear transition between low- and high-truncation regimes. This suggests that in the regime of close facet-to-facet interactions, molecular shape plays a primary role in determining the accessible phases, while the directionality of the interaction can be used to tune between the accessible phases for a given truncation level. We find that the formation of the different observed phases can be described as a trade-off between three main factors: minimising interparticle distance, maximising facet-to-facet contact, and maximising coordination number (Fig. 7(b)). A schematic representation of packing arrangements for hard particles and how well the competing factors are satisfied is given in Fig. S4 and dot plots for individual phases are given in Fig. S5. The balance between these trade-offs depends on the truncation level and directionality of interactions. Given the marked differences observed across truncation levels, we split our analysis by truncation and examined how varying the interaction directionality affects the resulting packing behaviour in each regime.

In simulations of hard truncated octahedra under high pressure with no directional interactions, only a single phase forms at low levels of truncation (*t* = 0−0.2): the Minkowski lattice. In contrast, our simulations show that introducing directional interactions results in four distinct phases as a function of interaction directionality. At low *σ*_ang_ (high directionality), a diamondoid-like phase forms (dark blue in Fig. 6(a)). In this structure, the (truncated) octahedra exhibit 4-fold facet-to-facet coordination, forming a network reminiscent of that seen in POCs such as CC3^35^ (Fig. 6(bi)). Despite each cage having eight potential interaction facets, the combination of strong directionality and the low level of truncations leads to neighbouring cages sterically blocking alternate facets in order to maximise contact area (Fig. S6), restricting coordination to four.

As *σ*_ang_ increases (i.e., directionality decreases), there is a transition to a different phase (Fig. 6(bii)) that is similar to the BCC phase (Fig. 5). However, unlike ideal BCC packing, the polyhedra are slightly offset from the centre of the facets to try to accommodate both the directional interactions and the preferred inter-particle distance of 0.56 Å, all without any overlap of the hard polyhedra. This offset of the polyhedra blocks access to 2 of the facets on opposite sides of the octahedra, resulting in 6-fold coordination rather than the 8-fold coordination typical of BCC. This increase in the coordination number from four to six between the dark blue and dark green phases comes at the expense of complete facet-to-facet alignment, resulting in a lower degree of contact than the dark blue phase (Fig. S6), reflecting a trade-off between the interaction directionality and packing efficiency (Fig. 7(b)).

On further increasing *σ*_ang_, there is a transition to the light green phase (Fig. 6(biii)), and the coordination of the (truncated) octahedra again increases from 6 to 8. In this phase, the decreased directionality of interactions allows for a higher coordination number, with 2D layers of polyhedra with 6-fold coordination stacked directly on top of each other, leading to 8-fold coordination overall. However, this comes at the cost of facet-to-facet contact, with poorer alignment between the facets (Fig. 7(b)). Finally, the phase at highest *σ*_ang_, light blue in Fig. 6(a), contains two sublattices where there are facet-to-facet contacts only within each sublattice, but less directional interactions between the two sublattices (Fig. 6(biv)). This gives the same type of crystal connectivity reported to form in elemental lithium under high pressure^36^. This phase is not the Minkowski lattice as formed from simulations of hard octahedra in ref. ^34^, but is closely related to the BCC phase formed with densely packed octahedra, but with columns of atoms shifted. This structure forms over the denser Minkowski lattice due to the attractive nature of the octahedral facets, and this has also been observed for attractive octahedral nanocrystals^37^.

Our results show that changing the directionality of interactions between facets results in a large variation in structural phase behaviour. Notably, we do not observe the BCC phase expected for non-interacting octahedra at low density^28^, nor the Minkowski lattice seen under high pressure. This absence suggests that facet-specific interactions directly compete with the geometry of the nearly perfect octahedra, preventing the formation of the crystal structures typically observed for purely shape-driven systems. This is further supported by the sensitivity of the phase behaviour to small changes in *σ*_ang_ where minor changes lead to major structure shifts, indicating a strong competition between the polyhedral shape and directional interactions in stabilising the observed phases.

At high truncation (*t* = 0.6−1.0), only two phases occur as a function of interaction directionality. One phase (purple in Fig. 6(a)) is dominant for most values where *t* ≥ 0.6, which is a BCC phase where each truncated octahedra is coordinated by 8 others through the facets of the cage (Fig. 6(di)). This phase only forms when *t* > 0.4, as below that the vertices of the truncated octahedra block access to all the facets of the cage. When *t* > 0.4, the polyhedra are truncated enough that the vertices do not block access, resulting in 8-fold coordination even for low *σ*_ang_ (very highly directional interactions). As shown in Fig. S6 and Fig. 7(b), the high coordination does not lead to a significant decrease in the degree of contact between the facets, which is seen in phases at low *t*. Unlike in ref. ^34^, where the BCC phase appears only at a specific truncation value (*t* = 0.66), the BCC phase in our simulations emerges as the most dominant structure. This likely arises from the combination of the preferred inter-polyhedral distance and the directional interactions, both of which favour BCC packing over BCT or DBCT to maximise the facet-facet interactions. As a result, the BCC phase remains stable across a broader range of truncation values, rather than giving way to BCT or DBCT phases as seen in systems of non-interacting truncated octahedra^34^. Furthermore, in contrast to octahedra with low levels of truncation—where varying *σ*_ang_ produces a wide range of phases—the persistence of the BCC phase across all values of *σ*_ang_ suggests a synergistic relationship between the polyhedral shape and facet-to-facet interactions.

At higher truncation values (*t*), a second phase (Fig. 6(dii)) appears when the directionality of interaction is low. This phase is more disordered than the DBCT phase observed in high-pressure simulations of hard octahedra with similar truncation (Fig. 5)^34^. The disordered nature of this structure arises from a mismatch between the preferred facet-facet interaction distance in our model (0.56 Å) and the interparticle spacing required to stabilise the DBCT arrangement. In the DBCT phase, the imperfect facet alignment leads to a larger optimal separation than our model favours, resulting in frustration and structural disorder. According to our clustering algorithm used to group the different phases (see the Methods section), this disordered yellow phase is also identified at *t* = 0.3, *σ*_ang_ = 0.3. However, this is a misclassification caused by the emergence of a disordered structure with multiple grain boundaries in one of the simulations, possibly due to local competition between the dark blue, pink, and dark green phases.

To understand the changing phase behaviour between low and high truncation levels, we analysed the structures for values of mid truncation, i.e., between *t* = 0.3−0.5. At low *σ*_ang_ (high interaction directionality), we observe an abrupt transition from the diamondoid-like structure (dark blue) to BCC (purple) between *t* = 0.4 and *t* = 0.5. As *σ*_ang_ increases, the interactions become less directional, and a different phase emerges between the low-truncation and high-truncation regions (Fig. 6(cii)).

Structurally, the pink phase resembles the BCC-like dark green phase, featuring 6-fold coordination along six of the eight available facets. However, a key difference lies in the relative positioning of neighbouring polyhedra. In the dark green phase, the polyhedra shift off the central facet-to-facet position to balance the interaction directionality, preferred interparticle distance, and steric constraints imposed by the polyhedral shape. In the pink phase, increased truncation reduces shape frustration, resulting in smaller shifts and larger interparticle distances at a given *σ*_ang_ to balance these competing effects. Nevertheless, the outcome—6-fold coordination—is preserved. Interestingly, unlike the dark green phase, including next-nearest neighbours in the analysis reveals that the coordination environment is BCC.

At *t* = 0.5, the pink phase only appears when the interactions are sufficiently flexible (i.e., directionality is low) to permit slight off-centre alignments. At low *σ*_ang_, such shifts are energetically unfavourable, preventing this phase from forming. Counterintuitively, this leads to the purple BCC phase (Fig. 6(ci)), which has a higher coordination number (8), contrary to the trend observed at lower truncation levels where increasing interaction directionality typically reduces coordination. This increase in coordination arises from a competition between the highly directional interactions maximising the contact between facets, and the preferred interparticle distance of 0.56 Å set by *σ*_LJ_. Rather than forming the 4-fold coordinated structure seen in the dark blue phase at lower truncation, where particles can pack closely, the system instead adopts the 8-fold coordinated BCC phase at a larger interparticle distance (Fig. 7(a)). This greater distance accommodates the steric constraints due to the polyhedral shape, while maintaining high directionality between the facets.

*t* = 0.5 marks a tipping point where the energetic cost of increasing interparticle distance is outweighed by the energetic gain from additional facet-facet contacts. At this truncation level, the geometry permits neighbouring polyhedra to interact with all eight facets of the central particle, but only at a separation ≈0.1 Å greater than the equilibrium distance set by the Lennard-Jones potential, as closer contact would result in overlap between the polyhedral particles. As truncation increases beyond *t* = 0.5, this constraint is lifted, and close packing becomes possible without geometric frustration. This leads to the formation of the BCC phase at higher truncation levels with a shorter interparticle distance (Fig. 7(a)).

### Phase coexistence

At the boundaries between phases in our phase diagram (Fig. 6(a)), repeated simulations under identical conditions sometimes yielded different phases. This effect is particularly evident at low *t*, reinforcing the observation that facet-to-facet interactions compete with the underlying polyhedral shape in this region of parameter space. To determine whether multiple phases are energetically equivalent given the same input parameters (*σ*_ang_, *t*), we calculated the average potential energy per particle at each temperature step for each of the five simulation runs, and plotted it as a function of temperature for the cases that exhibited phase coexistence: (0.6, 0.0), (0.7, 0.0), (0.6, 0.1), (0.7, 0.2), (0.3, 0.3), (0.4, 0.3), (0.5, 0.3), (0.7, 0.3), (0.7, 0.4), (0.8, 0.4), (1.0, 0.5) (Fig. S7). In many of these cases, such as (0.6, 0.1), (1.0, 0.5), (0.6, 0.0), (0.5, 0.3), and (0.4, 0.3), the different phases showed nearly identical energies, suggesting they are effectively energetically equivalent under the given conditions. From a chemical perspective, such coexistence may help explain the polymorphism observed in MOCs synthesised under similar experimental conditions.

In the case of (0.3, 0.3), although the phase was initially classified as mixed, further analysis revealed that all clusters have the same coordination environment. The appearance of a different phase arose from the formation of grain boundaries in one simulation, leading to a more disordered RDF. These grain boundaries correspond to a higher energy configuration, likely resulting from the initial nucleation process. Similar effects were observed for other input parameters such as (0.7, 0.0) and (0.4, 0.3), where variation in energy between configurations forming the same phase could be attributed to grain boundary formation. An example of a grain boundary formed in the simulations is shown in Fig. S8.

There are, however, a few cases where the coexisting phases differ in energy by approximately 3 kJ mol^−1^particle^−1^, notably for a range of truncation values at *σ*_ang_ = 0.7 and at (0.8, 0.4) (Fig. S7). In these instances, a competition arises between the light blue and light green phases, with the light blue phase consistently exhibiting higher energy. The repeated observation of both phases suggests this is not a statistical outlier. We propose that the light blue phase is stabilised by greater configurational entropy, which makes it competitive despite its higher enthalpy, particularly given that Monte Carlo simulations sample both enthalpic and entropic contributions, allowing phases of comparable free energy to coexist.

### Implications for MOCs

In comparing the phases from Gantapara et al.^34^ to those from our simulations, we find parallels between two of the five phases: BCC-like (dark green and pink), BCC (purple), and DBCT-like (yellow). The directional interactions and small interparticle distance in our simulations mean that no distorted phases are seen, as they would lead to less directional interactions with a larger interparticle distance, which is why BCC is persistent for a much larger range of truncation values than in the study of purely hard octahedra.

To understand how the different solid-state structures formed may affect the properties of the MOCs, we looked at the extrinsic porosity within each of the clusters, using the process outlined in Section “Porosity measurements”. We found that only one of the seven phases leads to a large extrinsic pore (Fig. S9), the dark blue phase (Fig. 6). The dark blue phase occurs at low levels of truncation and high interaction directionality, and so the extrinsic porosity likely arises from the competition between the molecular shape and maximal facet-to-facet contact, which results in a low coordination.

Exploring the effect of truncation and interaction directionality can also give insights into the phase behaviour of MOCs in two key ways. (1) It reveals the extent to which phase behaviour is robust to small changes in the chemistry of the molecules or experimental conditions, as long as the dominant interaction between the cages (e.g., facet-to-facet) remains the same. (2) It highlights the synergy between molecular shape and dominant interactions. For instance, if a single phase consistently forms for a given truncation level, irrespective of the directionality of interaction, as is the case for high levels of truncation, the interactions between the molecule can be thought of as compatible with the shape. Conversely, if a given truncation level leads to a wide variety of phases depending on interaction directionality, as seen for smaller values of *t*, this suggests that the molecular shape and interactions are in competition, and a stronger driving force may be needed to stabilise a particular phase. For low to mid truncation levels, there are also comparatively more mixed phases—where simulations with the same parameters lead to different phases—than with high truncation levels. This variability implies a weaker synergy between shape and interaction as compared to high truncation results, which produce more consistent results.

The relevance of our coarse-grained simulations in capturing trends and explaining observations in real molecular systems is supported by previous studies on octahedral POCs^21^. In that work, we showed that the dominant packing motif determines whether a single phase forms consistently or multiple phases emerge as a function of interaction directionality. The results from the simulations performed in ref. ^21^ matched experimental observations, where only one phase has been reported for one packing type, while the other gives rise to a range of distinct crystal structures. We therefore suggest that the insights gained here may help elucidate how shape and interactions interplay in the crystalline phase behaviour of MOCs.

The question remains how we might use chemical features of molecules to navigate the phase space explored by changing shape and interaction directionality. MOCs in particular offer an inviting playground for tuning the chemistry of polyhedral-shaped molecules, due to the wide variety of metal ions and linkers available for their assembly. The truncation of MOC polyhedra can be changed through the design of the linker, by altering the shape and connection between metal nodes, or by modifying the steric bulk surrounding them. From our previous work on POCs^21^, we have shown that increasing the steric bulk changed the directionality of interactions, making them more directional as it increases the energetic barrier of offsetting the facets of the cages. This steric effect likely contributes to the high directionality seen in the MOC [Rh_12_(NDI-Phe)_12_], which has steric bulk around the metal nodes emanating from the phenyl groups within the organic linker.

Interactions between the building blocks of MOCs also strongly influence the directionality of intermolecular interactions. For example, weak van der Waals forces typically result in low-directionality interactions, whereas strong forces such as intermolecular hydrogen bonds between linkers on neighbouring cages can introduce greater directionality, even in the absence of high steric bulk. What remains less well understood is how to rationally design specific packing motifs, such as facet-to-facet arrangements. As a result, the link between molecular building blocks and design rules for solid-state packing is still not fully established. Dimer calculations offer a promising route to clarify this relationship. This is a line of research we are actively pursuing, with the goal of establishing concrete design rules, not only for how changes in chemical functionality influence shape, interaction, and packing behaviour, but also for how to target specific packing motifs.

### Comparison to known phases

**Fig. 8:**
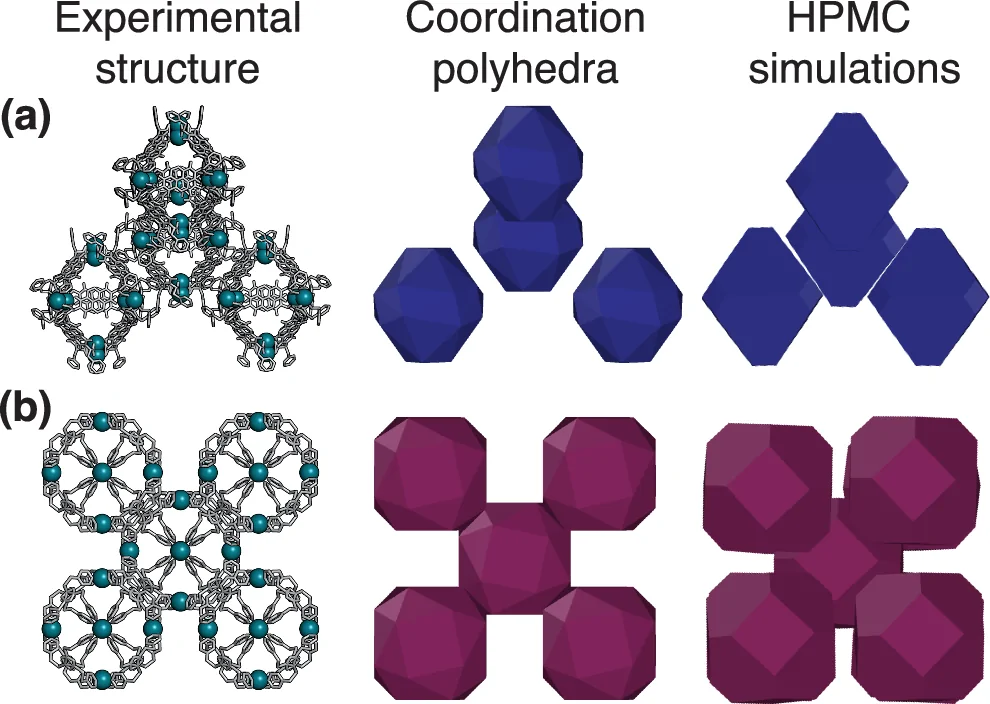
The match between experimentally reported crystal structures and the results from our simulations. **a** The MOC [Rh_12_(NDI-Phe)_12_(pyridine)_6_] forms the diamondoid structure WaaF-1 (CCDC code 2321951)^24^, which matches the dark blue phase in our phase diagram. **b** The MOC [Pd_6_(tpst)_8_]^12+^ forms a BCC structure (ROMYUQ)^39^, matching the purple phase in our phase diagram. All bonds are shown in grey, and the metal ions are shown in teal.

Running the Monte Carlo simulations can give an idea of how shape and interactions interplay in the solid-state phase formation of cage-like molecules. Matching these up with dimer calculations of a cage then informs what crystal structure is likely to form. From the dimer calculations on [Rh_12_(NDI-Phe)_12_(pyridine)_6_] and its shape, we would expect the phase behaviour of WaaF-1 to occur at high directionality (low *σ*_ang_)—based off the preferences seen in the dimer calculations that optimised to minimal deviation from the centroid-to-centroid axis—and a truncation level of *t* = 0.3−0.4, which corresponds to the dark blue phase in Fig. 6. Whilst the dimer calculations can indicate whether *σ*_ang_ is qualitatively low or high, we cannot yet assign an exact value because *σ*_ang_ remains an effective coarse-grained parameter whose quantitative mapping to atomistic dimer energy barriers has not yet been established. Comparing the experimentally reported crystal structure to this phase, we find our simulations were able to accurately predict the crystal structure (Fig. 8(a)). Highlighting how dimer calculations in combination with HPMC simulations can be used for CSP.

We also sought to evaluate how the phases observed in our simulations compared with crystal structures other than [Rh_12_(NDI-Phe)_12_(pyridine)_6_] that are reported in the literature. As we already had an example of a phase with low truncation, we were particularly interested in finding an example with a higher level of truncation. To find an example of a truncated octahedron with directional interactions between the facets, we looked through the structures reported by Li et al.^38^ and found the MOC [Pd_6_L_8_]^12+^ (L = 2,4,6-tri[(4-pyridyl)sulfanylmethyl]-1,3,5-triazine (tpst)) which crystallises into the cubic space group $Pn\bar{3}n$ with CCDC code ROMYUQ^39^. Unlike for [Rh_12_(NDI-Phe)_12_(pyridine)_6_], we did not perform dimer calculations on the structure from Xue et al.^39^, as the MOC is charged and the results would be dominated by the electrostatic interactions in the absence of counterions, conditions that do not accurately reflect the solid-state phase behaviour. Instead, we compared the experimentally reported crystal structure to the phases from our simulations, and found a match to the purple phase in Fig. 6, which packs in a BCC arrangement (Fig. 8(b)). Given that the cage exhibits a high level of truncation (*t* ≈ 0.8) and packs in a facet-to-facet manner, this result is consistent with our phase diagram that indicates that this phase is robust across the full range of interaction directionalities and a large range of truncation levels.

### Outlook

We have presented an approach for predicting the packing behaviour of charge-neutral MOCs using a multiscale modelling technique that combines atomistic calculations with coarse-grained Monte Carlo simulations. As a proof of concept, we focused on the MOC [Rh_12_(NDI-Phe)_12_(pyridine)_6_], as reported by Tokuda and Furukawa^24^. The dimer calculations indicated a favourable facet-to-facet packing, which was used to inform a coarse-grained model. We then employed this model to explore how molecular truncation and interaction directionality affect the solid-state phase behaviour of pseudo (truncated) octahedral MOCs.

Our dimer-informed coarse-grained simulations successfully reproduced the experimentally observed structure of [Rh_12_(NDI-Phe)_12_(pyridine)_6_], and changing the directionality of interaction and extent of truncation of the octahedra leads to six additional phases, one of which matches another MOC reported in the literature. The dimer calculations, performed with implicit solvation, captured the observed facet-to-facet packing behaviour, which allowed the experimentally reported WaaF-1 structure to be recovered in the coarse-grained simulations. The equivalent vacuum calculations show the same qualitative energetic ordering, indicating that this packing preference is robust rather than uniquely stabilised by an implicit representation of ethanol. Therefore, while the present calculations do not address the full solvent-induced pseudopolymorphism reported experimentally, which would require explicit treatment of DMA and specific solvent-cage interactions, they show that implicit solvation can be incorporated into the dimer-informed workflow while retaining the ability to predict the experimentally observed WaaF-1 packing motif. Combined with the efficiency of the HPMC simulations, this workflow offers a realistic route to high-throughput screening of MOC phase space to identify candidates with desirable properties, such as high porosity.

This work demonstrates a CSP workflow for MOCs that combines dimer calculations with coarse-grained simulations. While the approach is currently best suited for neutral cages where counter-ions do not dominate the interaction landscape, neutral MOCs represent a significant proportion of reported MOCs. Moreover, the relatively strong bonds between metal ions and anionic ligands (compared to neutral ligands) often enhance their solid-state stability^40^. As we have shown previously^22^, coarse-grained simulations can be used to construct plausible crystal structures for subsequent optimisation. These optimised structures can then be compared to experimental data to aid in characterisation. Therefore, even for charged systems where dimer calculations may be unreliable, counterions could be added to atomistic structures generated from coarse-grained models with a range of input parameters before further optimisation and comparison to experimental diffraction data. Matching the predicted structure to experimental data eliminates the need for single-crystal growth, which is often a barrier to solid-state characterisation^41^.

Beyond structure prediction, our coarse-grained model offers insights into the driving forces behind phase behaviour. By varying the model parameters, we explored the interplay between molecular shape and interaction in phase formation. We find that cages with truncation levels greater than *t* = 0.5 exhibit a more synergistic relationship between their shape and interaction, leading to more consistent phase behaviour, while lower truncations result in greater sensitivity to interaction directionality. Looking ahead, dimer-informed coarse-grained models provide an opportunity to explore chemical space quickly, opening up the rational design of solid-state MOCs with tailored properties.

## Methods

### Dimer calculations

Dimer calculations were carried out using GFN2-xTB^25^, as it has been shown to perform well when looking at metal-containing species, including MOCs^42,43^. The molecule was extracted from the crystal structure reported by Tokuda and Furukawa^24^, but could be made using software such as stk^10^ in the future if required. Although this could bias the results, we noticed a large rearrangement in the orientation of the phenyl rings on relaxation, which likely removes the problem of structural biasing.

The molecule was geometry optimised with the optimisation level set to tight, corresponding to convergence thresholds as laid out in Table 1 using stko^44^. With the optimised cage, we created dimers to examine the energetics of high-symmetry packing motifs: facet-to-facet, facet-to-vertex, edge-to-facet, edge-to-edge, and vertex-to-vertex (see Fig. S1). Each dimer was made using the package https://github.com/ewolpert1/dimer_calculations which is built on stk (https://github.com/lukasturcani/stk)^10^, where a dimer is formed by displacing a second cage along the axis from the centroid of the first cage to the centroid of its facet/vertex/edge. For each dimer, we checked whether the cages were catenated or had atomic overlap before optimising the dimer. Dimers that were considered catenated or had overlapping atoms were removed before the calculations were performed (Supplementary Section 11).

**Table 1:** Optimisation levels in xTB and corresponding convergence thresholds for energy (Econv) and gradient (Gconv)

Optimisation level	Econv/E_h_	Gconv/E_h_*α*^−1^
crude	5 × 10^−4^	1 × 10^−2^
sloppy	1 × 10^−4^	6 × 10^−3^
lax	2 × 10^−5^	2 × 10^−3^
normal	5 × 10^−6^	1 × 10^−3^
tight	1 × 10^−6^	8 × 10^−4^

To ensure adequate configurational phase space was explored, the relative displacement and rotation of the two cages were varied. This varied (i) the angle of rotation of the second cage around the displacement axis (0^∘^, 30^∘^, 60^∘^, 90^∘^), (ii) the displacement of the second cage perpendicular to the displacement axis. This off-centre displacement was only investigated for facet-to-facet, edge-to-facet, and edge-to-edge interactions, and was determined by taking the size of the cage *d* and creating a circle of diameter *d* perpendicular to the displacement axis, then translating the centroid of the second cage by sampling points at integer intervals within the circle. (iii) The distance between each cage. The initial distance between the two cages was set by placing the centroid of the displaced cages at twice the distance from the centroid to the edge of the cage (e.g., centroid-facet or centroid-vertex), minus 2 Å. For subsequent calculations, the cage was incrementally displaced outward in 1 Å steps. This process continued until no overlaps were observed between the cages for any given rotation or perpendicular displacement at a given separation.

The geometry optimisations were first performed with constraints between all metal-metal ions in the dimer. This was to ensure the optimisation did not change the relative positions of the two cages in the dimer to get a sense of how directional the interactions are. To assess the quantitative accuracy of the GFN2-xTB interaction energies, we calculated the absolute interaction energy of the lowest-energy dimer in vacuum. Geometry optimisations were performed on dimers in the gas phase using the optimisation levels “crude”, “sloppy”, “lax”, and “normal”, where dimers within the lowest 100 kJ mol^−1^ were taken forward for calculations at stricter optimisation levels, as well as the lowest-energy dimer for each packing type if none were found within 100 kJ mol^−1^. We then performed a final optimisation, removing the constraints between metal-metal ions, confirming the lowest-energy dimer was facet-to-facet with an interaction energy of −350 kJ mol^−1^. Comparing this to the −433 kJ mol^−1^ reported by Tokuda and Furukawa^24^ using LRC-*ω*PBE/6-311G with many-body dispersion correction shows that although GFN2-xTB underestimates the magnitude of the interaction by approximately 80 kJ mol^−1^, it captures the same qualitative conclusion that the facet-to-facet dimer is strongly stabilised.

To determine the effect of solvation, further calculations with implicit solvation were performed using the analytical linearised Poisson-Boltzmann ALPB model in GFN2-xTB^27^. The partial charges used by ALPB were those generated internally by the GFN2-xTB calculation for each atomistic structure. The solvent dielectric constant and non-electrostatic ALPB parameters were taken from the built-in xtb/ALPB solvent parameter set for ethanol, corresponding to reference parameters used by the xTB implementation. The solvated dimer calculations took dimers from the initial “crude” gas-phase calculations, which were <150kJ mol^−1^ higher in energy than the lowest-energy dimer, and optimised them with the added implicit solvation. We also optimised the lowest-energy dimer for each packing type, even if it was more than 150 kJ mol^−1^ higher in energy, to ensure no packing arrangements that may be stabilised by solvation were missed. The calculations with implicit solvation were first performed using the optimisation level “crude”, and further calculations were performed using optimisation levels “sloppy”, “lax”, and “normal”, where dimers within the lowest 100 kJ mol^−1^ were taken forward for calculations at stricter optimisation levels, as well as the lowest-energy dimer for each packing type if none were found within 100 kJ mol^−1^. We then performed a final optimisation, removing the constraints between metal-metal ions, and calculated the RMSD between the facet-to-facet dimers which were within 100 kJ mol^−1^ of the lowest energy configuration using the RMSD Python package^45^, which implements the Kabsch algorithm^46^ and the Hungarian algorithm^47^. Alignments were performed excluding hydrogen atoms, coordinated pyridines, and flexible phenyl groups.

### Hard particle Monte Carlo simulations

Monte Carlo simulations were performed using HPMC^32^, a plugin for the HOOMD-Blue simulation toolkit^33^. The HPMC simulations employ the Metropolis Monte Carlo algorithm, where trial moves are either translations or rotations of the hard particles. A move is accepted if it does not produce any overlaps between particles, whereas if an overlap occurs the move is rejected. As a result, these simulations are often considered temperature-independent, since moves either result in no energy change or infinite energy increase^32^. However, in our simulations, we included additional interactions between neighbouring particles. These interactions modified the acceptance criteria (see Eqns. (1–4)), introducing energetic penalties for moves based on an interaction potential, making the results temperature-dependent.

Simulations were performed in the *NVT* ensemble and started at *k*_B_*T* = 1.35 before being gradually cooled through the transition point *k*_B_*T* ≈ 1. The temperature was reduced according to *T*_*i*+1_ = 0.95*T*_*i*_ where *k*_B_ = 0.00831 kJ mol^−1^ K^−1^. Each simulation ran for 24 hours using 64 cores on the ARCHER2 UK National Supercomputing Service^48^. At each temperature, 10^8^ timesteps were performed, where one trial move is applied to a random number of particles in each cell during one timestep. The computational time per timestep varied with the number of interactions calculated. As polyhedra aggregated into clusters, the time per timestep increased. Consequently, due to the finite wall time, some simulations—particularly those at low *σ*_ang_—completed fewer temperature points. For these cases, once a simulation concluded, the final configuration was taken as representative of the phase behaviour at lower temperature.

Simulations were performed with 512 particles in cubic boxes with periodic boundary conditions measuring 12 Å per side. The hard octahedral particles before truncations were unit length. Since the potentials were attractive and the systems were initialised at low density, the polyhedra assembled into compact clusters without mechanical stresses and structural defects, which are often seen in bulk simulations.

### Phase analysis

As in ref. ^21^, we have chosen to determine the phase diagram based on the RDFs containing only the central positions of the cages, where the RDFs were measured between 0 and 1.9 Å. This loses some information, as we exclude the orientational behaviour of our structure, but all phases were visually inspected to ensure there were no differences. The phases were analysed by clustering the similarities between the RDFs as performed by dynamic time warping^21,49^. The code used to determine the similarities can be found at https://doi.org/10.5522/04/29597369. We clustered the data into seven separate clusters, as that was the minimum number required to separate the six phases we could determine by eye. We used a Gaussian filter of sigma = 3 to smooth the RDF in order to directly compare the phases with larger noise due to less stringent parameters i.e., higher values of *σ*_ang_, to phases where there is less noise. This allowed for the determination of structural similarities regardless of the values of *σ*_ang_. The contact area between the facets was calculated using the facet_overlap.py script found at https://doi.org/10.5522/04/29597369 with the variables max-gap = 0.1 and max-angle = 10. This calculated the intersection area between triangular facets on adjacent polyhedra, which was divided by the total area of the triangular facet. The values were averaged over all particles and configurations for a given combination of *t* and *σ*_ang_.

To classify the phase behaviour of different clusters, we used the polyhedral template matching modifier^50^ in OVITO^51^ to determine the different structural environments. This highlighted the BCC crystal structures for both the pink and purple phases. For non BCC phases, we looked at the nearest-neighbour shell, contact area between facets, and RDFs to analyse the packing arrangement in terms of the coordination number and relative orientation.

### Porosity measurements

To determine the extrinsic porosity of the different phases, we used a custom-made script found at https://doi.org/10.5522/04/29597369 to take the output files from the HPMC simulations and convert the final frame to a CIF file. This script converted the hard polyhedra to atomic structures, scaling the unit-size octahedra such that the distance from the centroid of the cage to a vertex with no truncation was 10 Å. This atomistic structure has Li atoms at the vertices of the polyhedra and midpoints of the facets, and Cs atoms at the centre of the polyhedra. Cs atoms were chosen due to their large radii to fill the majority of the intrinsic porosity of the cage so it wouldn’t count toward porosity measurements. Li atoms were chosen due to their small radii to avoid any major obstruction of the extrinsic porosity measured. The script also condensed the simulation box size to be as small as possible around the cluster.

Using the created atomistic structure, we then calculated the pore size distribution within the structures using Zeo++^52^. The pore size distributions were calculated using the high accuracy flag with 50,000 Monte Carlo samples and a probe diameter of 1.2 Å.

### Computational cost

To estimate the computational cost required for CSP of a MOC using our workflow, we calculated the CPU-hours required for both the dimer calculations and HPMC simulations. We note that these values should be regarded as an upper estimate for the present proof-of-concept implementation, as the cost of dimer calculations could be reduced by optimising the sampling procedure, and the cost of the HPMC simulations could be reduced by employing an adaptive cooling rate. For example, the cooling rate could be adjusted based on the heat capacity, allowing the system to cool slowly through the phase transition but more rapidly away from the transition temperature.^53^

The total cost of the dimer calculations used to identify the low-energy implicitly solvated dimers across the different constrained/unconstrained and optimisation conditions was 62,117 CPU-hours. For the HPMC simulations, each simulation run required 24 h using 64 cores, corresponding to 1536 CPU-hours. Therefore, the approximate computational cost for predicting one crystal structure using a single HPMC simulation is ≈ 64,000 CPU-hours. More conservatively, accounting for multiple simulations across a range of input parameters and repeated runs for each parameter set, the total cost may be closer to 100,000 CPU-hours.

For context, Pulido et al.^54^ reported that determining the crystal energy landscape of T2E, an extended triptycene trisbenzimidazolone analogue, took ≈ 400,000 CPU-hours. A direct comparison between the two studies is necessarily approximate, since the systems differ in size, flexibility, chemical composition, solvent environment, and level of theory/parameterisation. In particular, the system studied here is a MOC with 822 atoms, whereas T2E is a purely organic molecule containing 64 atoms. Nevertheless, this comparison indicates that the dimer-informed coarse-grained workflow can provide access to crystal packing predictions for larger and more complex cage-like systems at a reduced computational cost relative to conventional CSP studies.

## Supplementary information

supplmentary information

Inventory of supplmentary information

Transparent Peer Review file

## Source data

Source Data

## Data Availability

The coarse-grained simulation data generated in this study are publicly available in the UCL Research Data Repository at https://doi.org/10.5522/04/29597369. The previously published crystallographic data used in this study are available from the Cambridge Structural Database under accession codes 2321951 (desolvated WaaF-1) and ROMYUQ ([Pd_6_(tpst)_8_]^12+^). Source data are provided with this paper.

## Code availability

The analysis code used in this study is publicly available through the UCL Research Data Repository at https://doi.org/10.5522/04/29597369. The custom dimer-calculation code used in this study (version 1.0.0) is archived on Zenodo at https://doi.org/10.5281/zenodo.21993197 and the repository is available at https://github.com/ewolpert1/dimer_calculations.
