# Supplementary material for: The role of shape and interaction directionality in the crystalline phase behaviour of octahedral metal-organic cages
Source: Nat Commun. 2026 Sep 22;17:11044. doi: 10.1038/s41467-026-77857-7 (PMC13690734; doi:10.1038/s41467-026-77857-7)
Supplement: Supplementary file 1 [file 41467_2026_77857_MOESM1_ESM.pdf]

Supplementary Information: The role of shape and interaction directionality in the crystalline phase behaviour of octahedral metal-organic cages

Emma H. Wolpert[1,2,3],* Andrew Tarzia[4,5], Yuhang Zhu[6], and Kim E. Jelfs[6]

[1]*Department of Chemistry, University College London, London, WC1H 0AJ, UK*

[2]*Department of Materials and the Thomas Young Centre for Theory and Simulation of Materials, Imperial College London, London, SW7 2AZ, UK*

[3]*I-X Centre for AI in Science, Imperial College London, White City Campus, London, W12 0BZ, UK*

[4]*Department of Applied Science and Technology, Politecnico di Torino, Corso Duca degli Abruzzi 24, 10129 Torino, Italy*

[5]*School of Chemistry, University of Birmingham, Edgbaston, Birmingham, B15 2TT, UK*

[6]*Department of Chemistry, Imperial College London, Molecular Sciences Research Hub, White City Campus, Wood Lane, London, W12 0BZ, UK*

E-mail: e.wolpert@ucl.ac.uk

Contents

1 Parameters for the coarse-grained model **S-3**

2 Dimer configurations **S-4**

3 Relative energy of dimers **S-5**

4 Low energy constrained dimer **S-6**

5 Simulation clusters **S-7**

6 Examples of favoured packing arrangements **S-8**

7 Structural descriptors **S-9**

8 Contact area of neighbouring facets **S-10**

9 Energy vs $k_{\mathrm{B}}T$ for parameters leading to multiple phases **S-11**

10 Grain boundary **S-12**

11 Determining catenation and overlaps **S-13**

12 Pore size distribution **S-14**

References **S-15**

1 Parameters for the coarse-grained model

Table S1: Interaction parameters used in the HPMC simulations.

		σ_{ang}									
		0.1	0.2	0.3	0.4	0.5	0.6	0.7	0.8	0.9	1.0
t	0.0	84	65	57	50	43	37	32	31	30	28
	0.1	84	65	57	50	43	37	33	31	29	28
	0.2	83	65	57	50	42	37	33	30	29	27
	0.3	84	65	58	48	41	36	32	30	28	27
	0.4	84	65	54	46	40	35	31	28	27	26
	0.5	81	53	44	41	37	32	30	27	26	24
	0.6	69	46	40	36	34	31	28	26	24	23
	0.7	65	42	38	35	32	29	26	24	23	21
	0.8	63	42	36	34	30	26	24	22	20	20
	0.9	61	41	36	32	28	24	21	19	18	17
	1.0	60	41	33	30	26	22	18	16	15	14

2 Dimer configurations

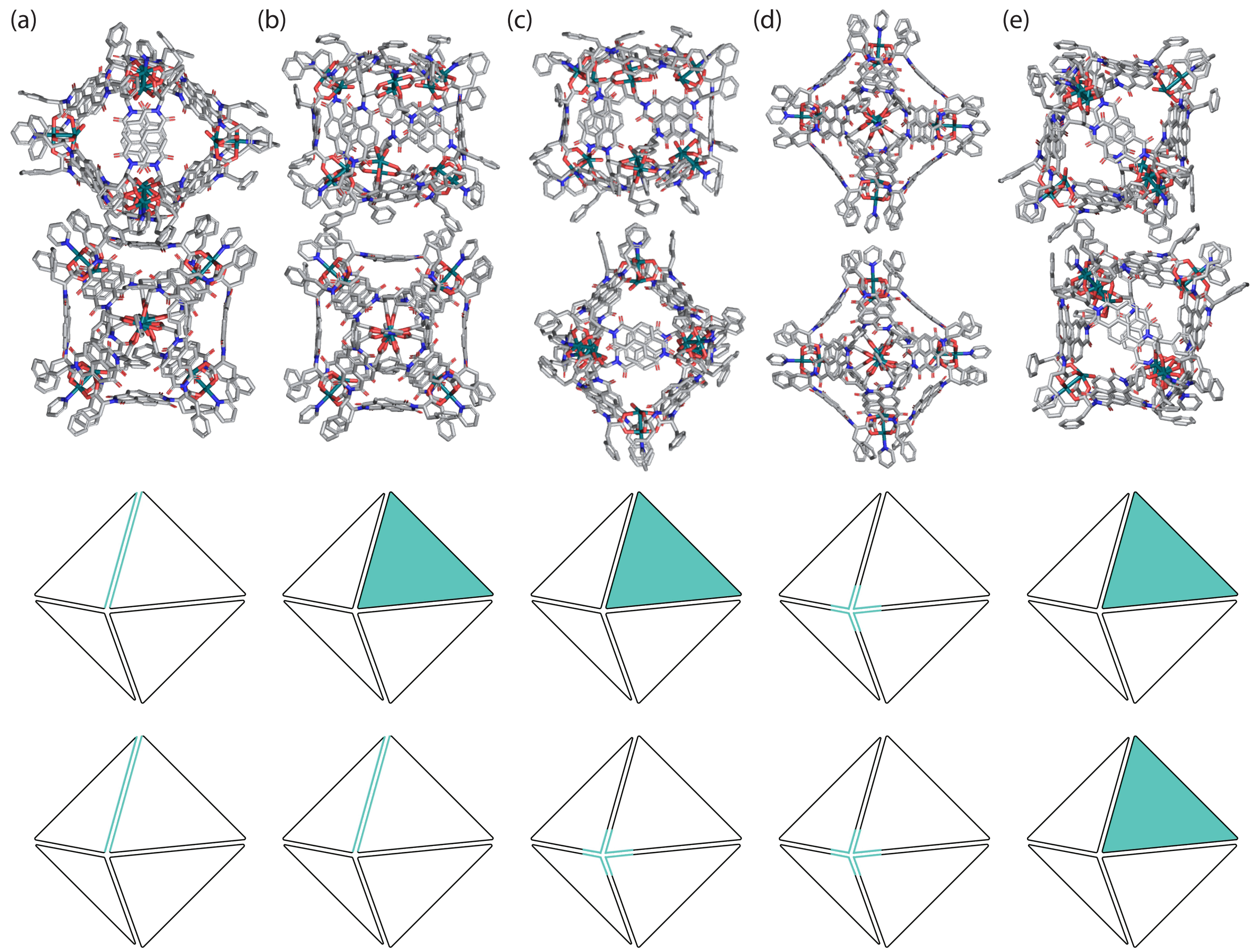

Figure S1: Examples of the different high symmetry dimers explored in this work. (a) edge-to-edge, (b) facet-to-edge, (c) facet-to-vertex, (d) vertex-to-vertex, and (e) facet-to-facet. Above is a representative configuration of the dimers formed by $[Rh_{12}(NDI\text{-}Phe)_{12}(pyridine)_6]$ where the bonds in the molecules are shown as grey, red, blue, and teal for carbon, oxygen, nitrogen, and rhodium respectively and hydrogens are omitted for clarity. Below is a schematic representation of the cage as an octahedron where the regions oriented towards each other in the calculations are highlighted in cyan.

3 Relative energy of dimers

Table S2: Energy of the lowest energy dimer for each packing type relative to the global lowest energy dimer for each optimisation threshold/constraint choice. The constraints refer to constraints between metal-metal distances in the calculation. Optimisation thresholds naming comes from xTB software as shown in Table 1.

	Relative energy ($kJ\,mol^{-1}$) for given solvent /metal-metal distance constraints /xTB optimisation threshold				
Packing type	Vacuum /constrained /crude	Ethanol /constrained /crude	Ethanol /constrained /normal	Ethanol /unconstrained /normal	Vacuum /unconstrained /normal
facet-to-facet	0	0	0	0	0
edge-to-edge	13.3	36.0	64.8	77.0	94.3
edge-to-facet	109.4	72.2	102.9	98.3	164.4
vertex-to-facet	113.2	98.6	129.7	194.9	227.4
vertex-to-vertex	179.1	107.2	139.7	195.5	298.9

4 Low energy constrained dimer

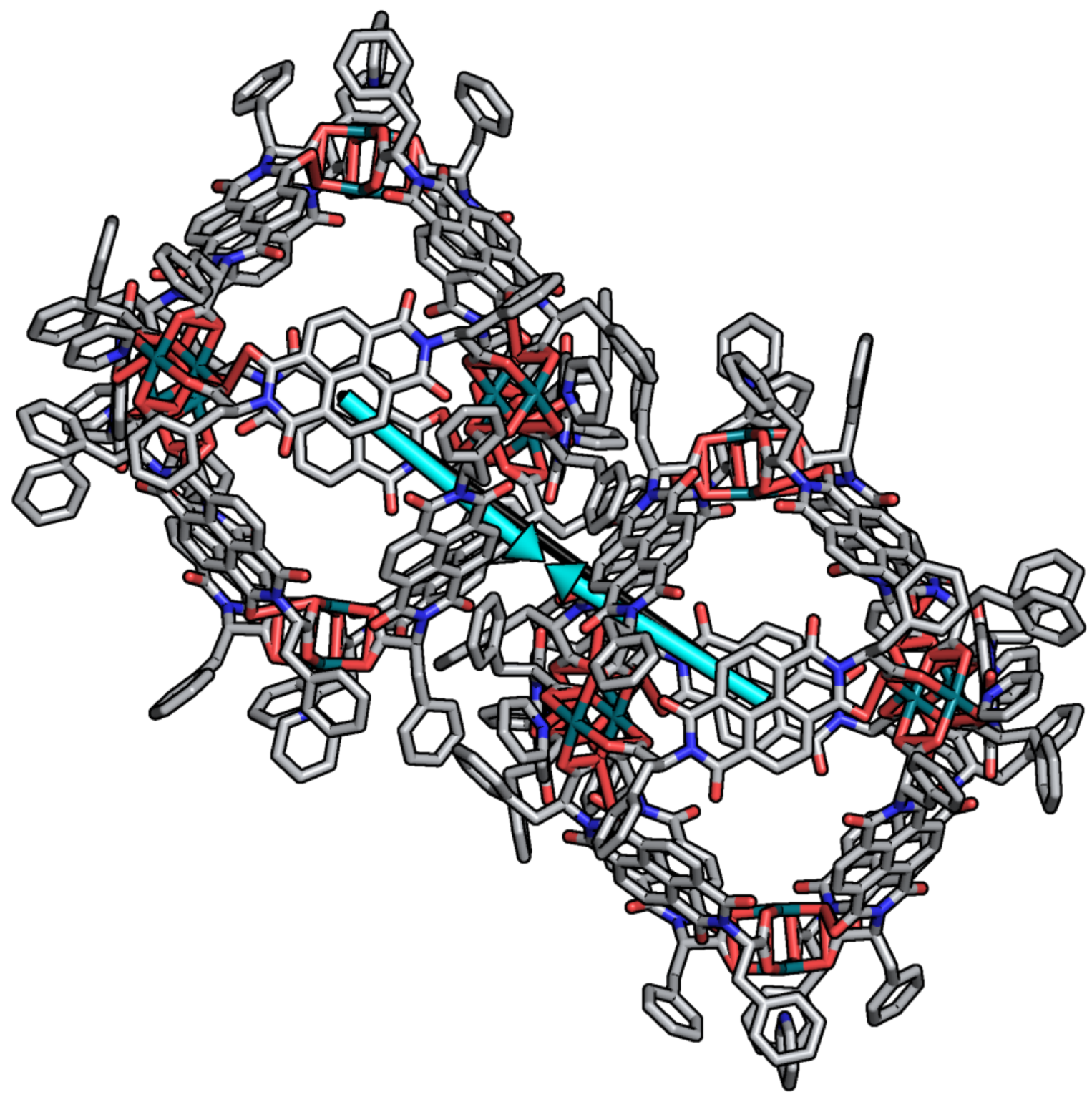

Figure S2: Lowest energy dimer of $[Rh_{12}(NDI\text{-}Phe)_{12}(pyridine)_6]$ with constraints between the metal-metal distances showing preferred facet-to-facet packing. The cyan arrows show the vector connecting the centroid of each cage to the centroid of the facet. The vector between the centroids of the two cages is shown in black. Bonds in the molecules are shown as grey, red, blue, and teal for carbon, oxygen, nitrogen, and rhodium respectively. Hydrogens are omitted for clarity.

5 Simulation clusters

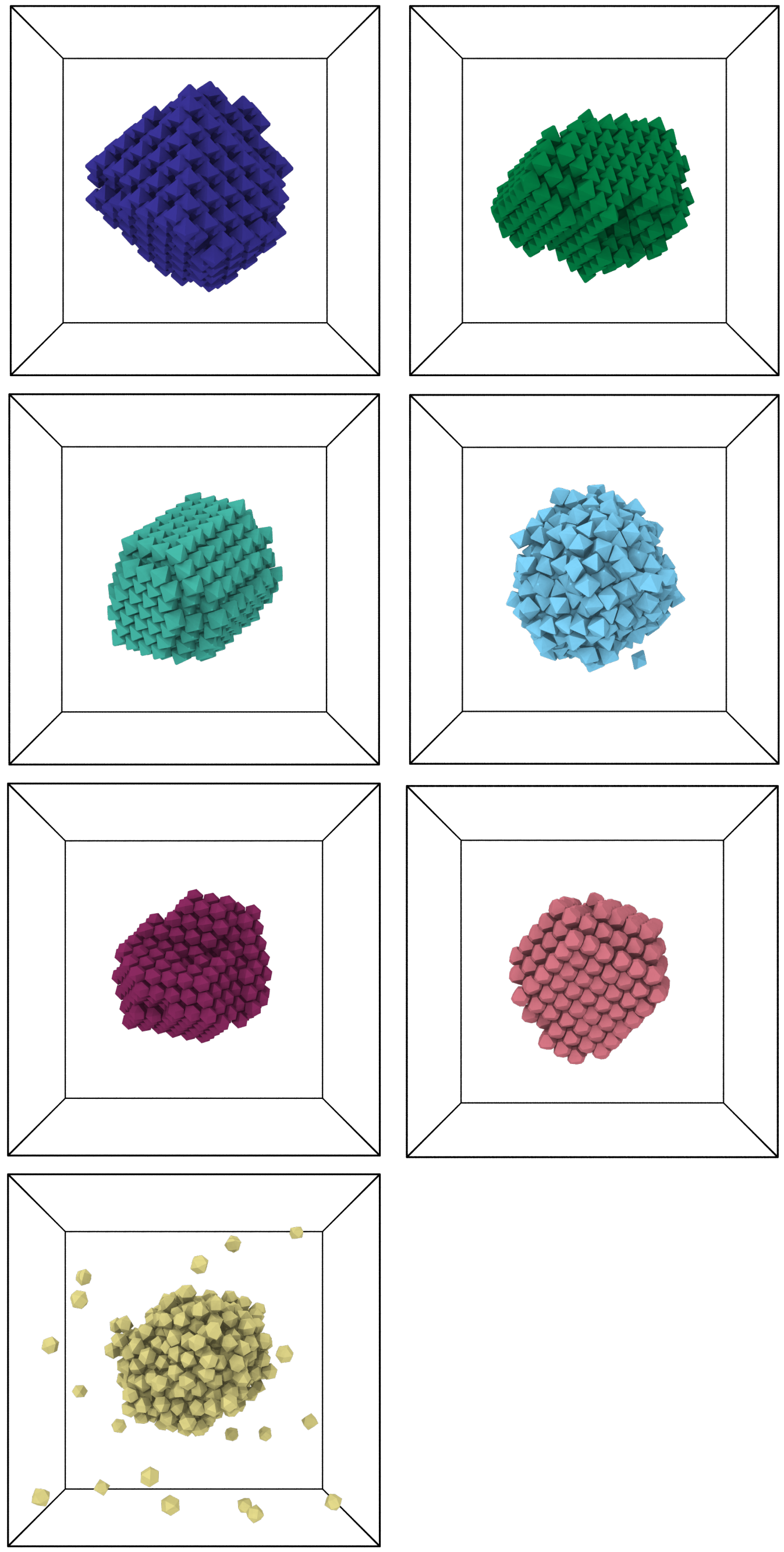

Figure S3: Representative clusters of different phases formed in the simulation. The colour of each cluster corresponds to the phase it represents in the phase diagram.

6 Examples of favoured packing arrangements

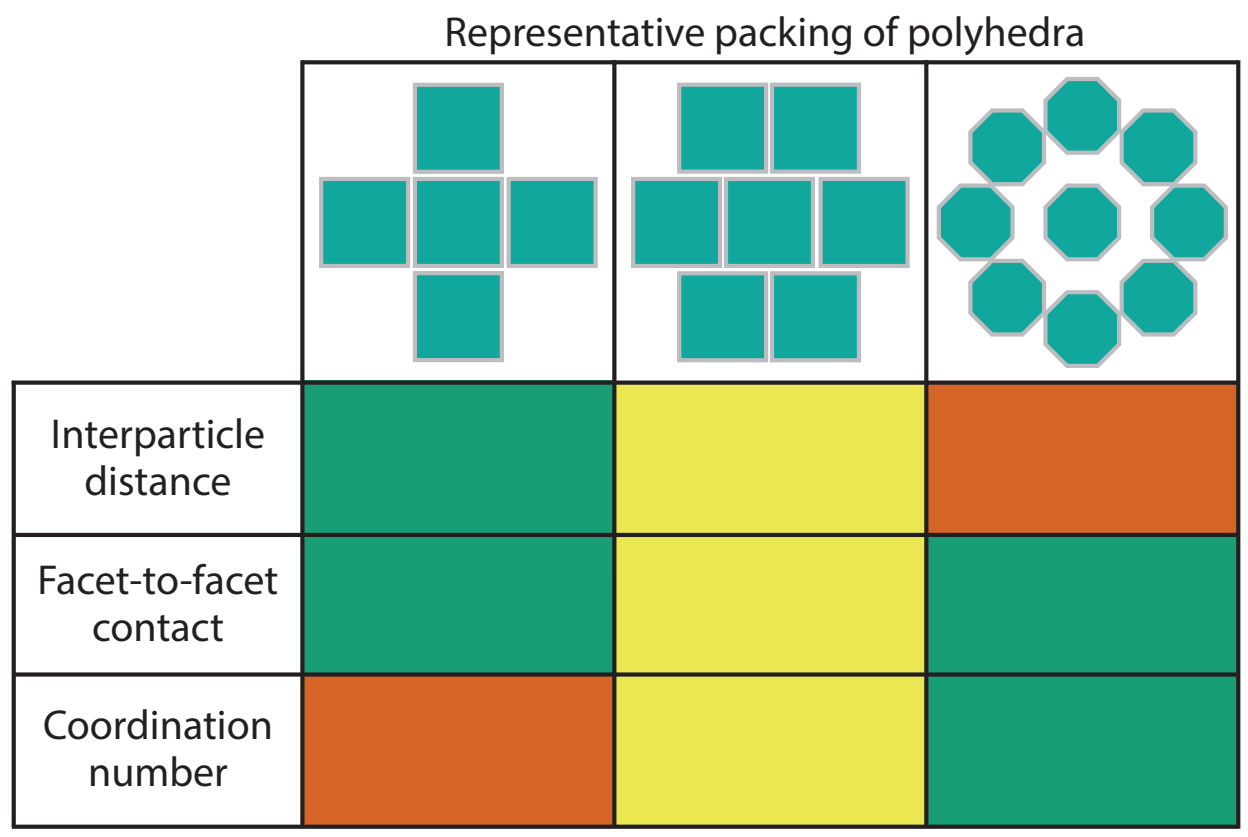

Figure S4: 2D schematic examples of different packing arrangements for hard particles. Green indicates that the criterion is well satisfied, yellow indicates that it is partially satisfied, and red indicates that it is poorly satisfied. For interparticle distance, “well satisfied” means close to the preferred interaction distance; for facet-to-facet contact, it means large facet overlap; and for coordination number, it means a high number of neighbouring particles.

7 Structural descriptors

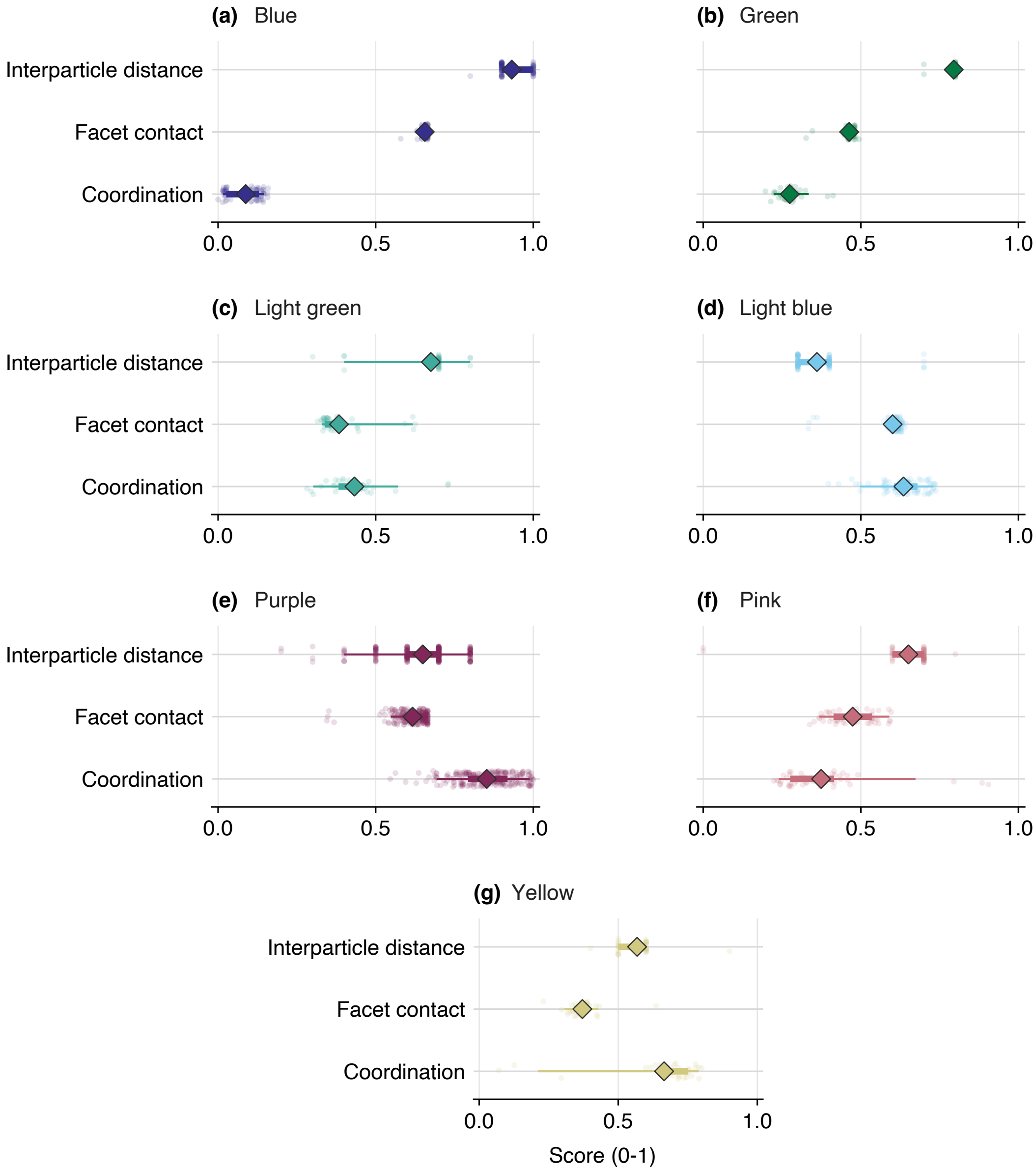

Figure S5: Phase-specific distributions of structural descriptors. Distributions of the interparticle-distance score, facet-contact fraction and coordination-number score are shown for the (a) blue, (b) green, (c) light green, (d) light blue, (e) purple, (f) pink and (g) yellow phases. Faint points represent individual simulations assigned to each phase. Thin horizontal lines indicate the 5th–95th percentile range, thick lines indicate the interquartile range and diamonds indicate the phase mean. The interparticle-distance and coordination-number scores are scaled from 0 to 1 across the complete dataset. The interparticle-distance score is inverted so that larger values correspond to shorter distances. The facet-contact fraction is intrinsically bounded between 0 and 1. Source data are provided as a Source Data file.

8 Contact area of neighbouring facets

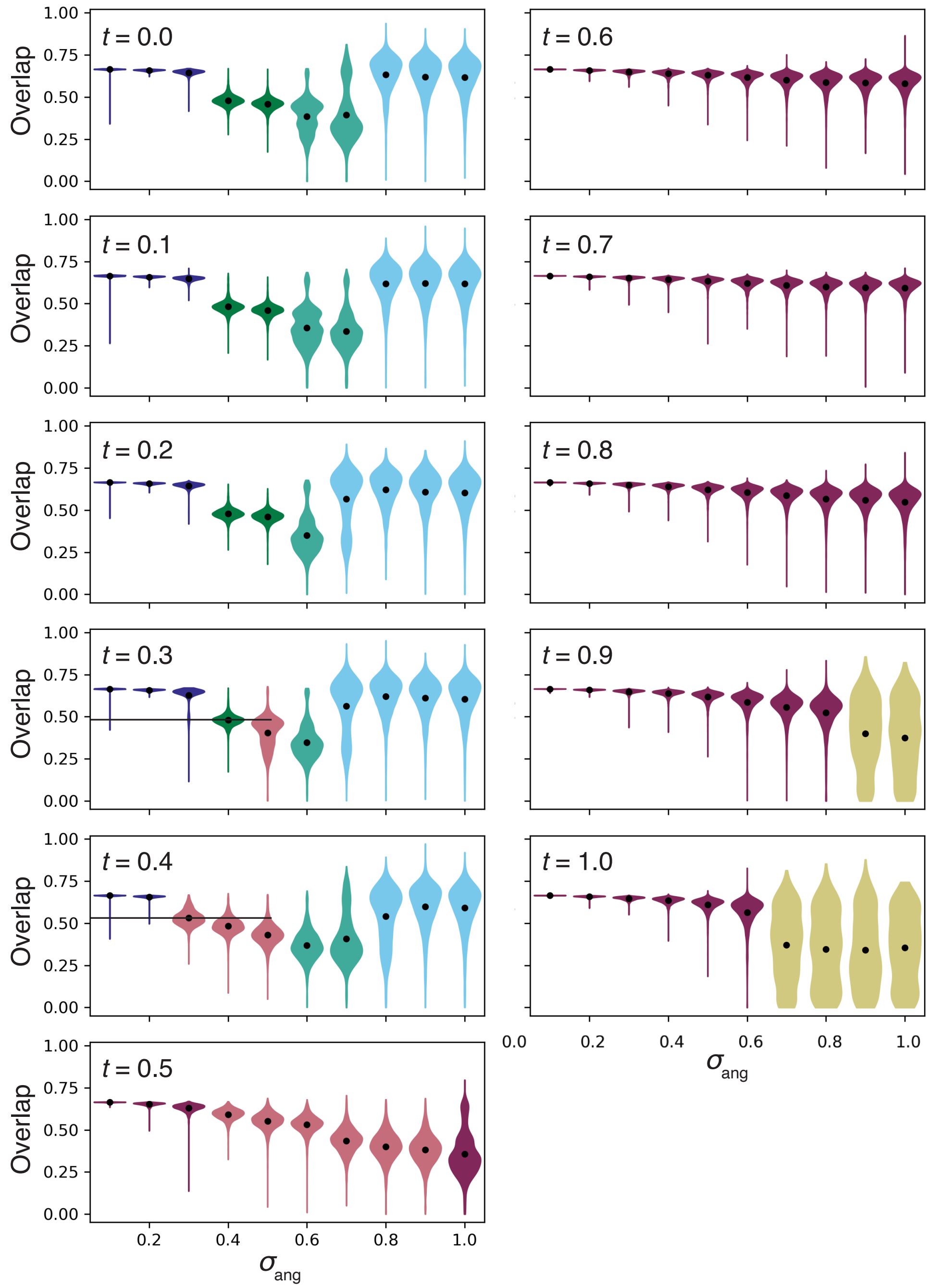

Figure S6: Violin plot of the contact area between facets of neighbouring polyhedra as a fraction of the total facet area for different values of σ_{ang}. Colours of the plot represent the phases that the majority of the configurations were assigned for a given combination of t and σ_{ang}. Source data are provided as a Source Data file.

9 Energy vs $k_\mathrm{B}T$ for parameters leading to multiple phases

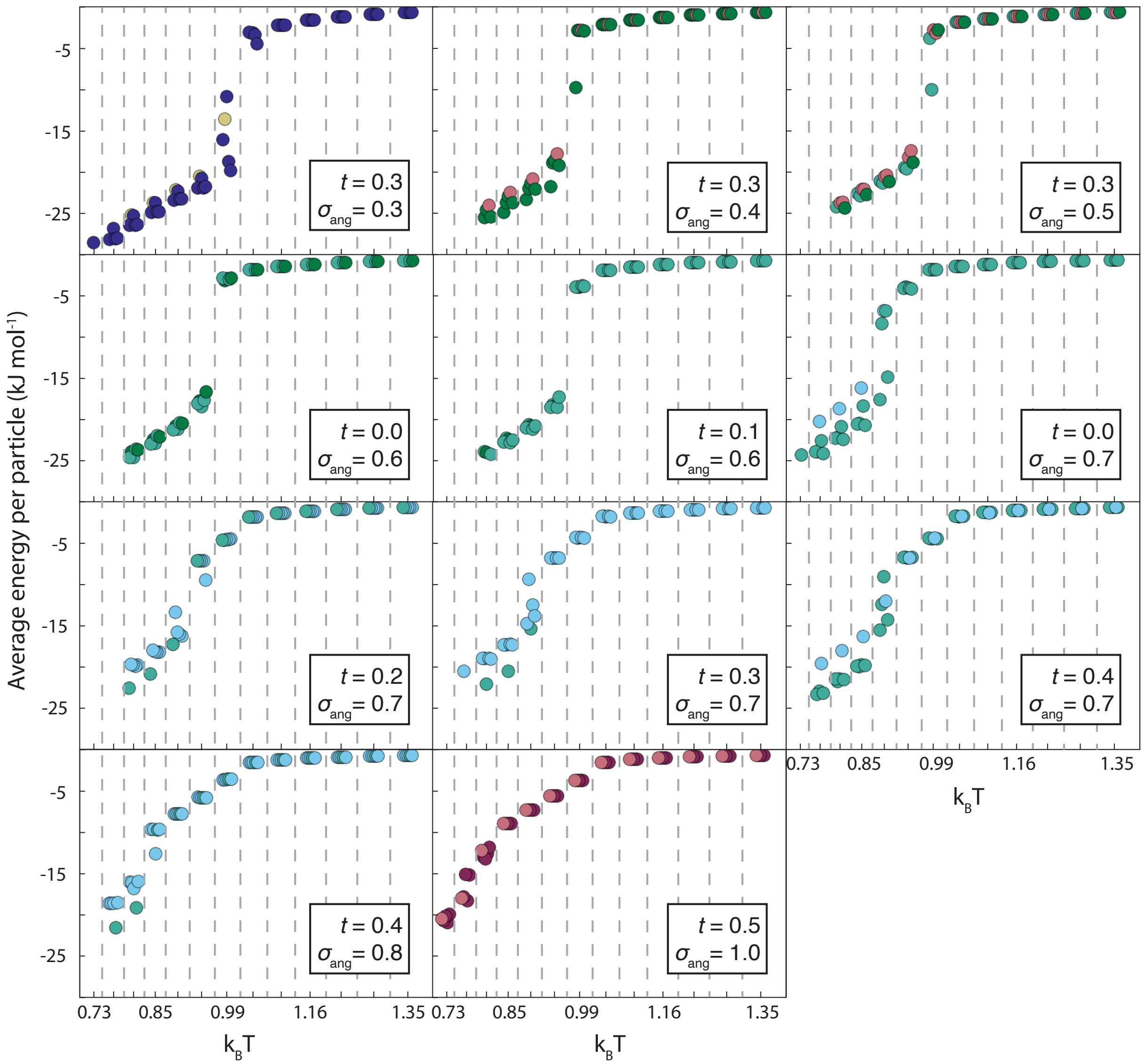

Figure S7: Plots of energy vs $k_\mathrm{B}T$ for each of the configurations at combinations of truncation t and σ_ang which lead to multiple different phases. Data points are coloured by their respective phases as shown in Fig. 6. Grey dashed lines are used to separate data points collected at different $k_\mathrm{B}T$. We note that not all values of $k_\mathrm{B}T$ have data points for each simulation run due to the simulations terminating after a finite wall time instead of after a certain number of moves. Source data are provided as a Source Data file.

10 Grain boundary

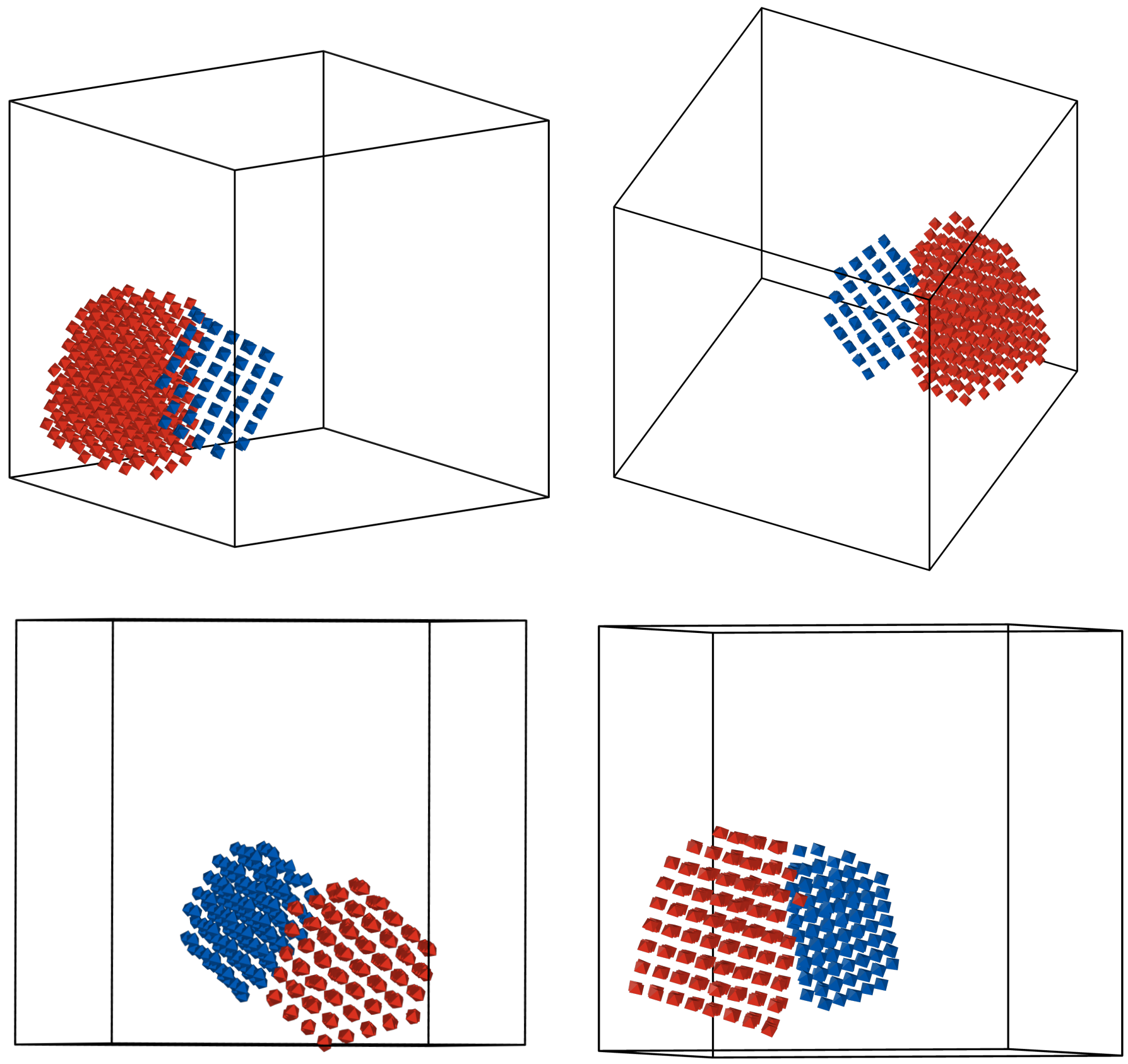

Figure S8: Different views of the grain boundary formed in the simulation where $\sigma_{\text{ang}} = 0.7$ and $t = 0$ where the two orientations of the crystals are coloured blue and red.

11 Determining catenation and overlaps

To check for atomic overlap, the code calculated distances between atoms in the two cages using RDKit's `rdMolTransforms.GetBondLength` function.[1] If non-hydrogen atoms were within 1 Å of each other, the molecules in the dimer were considered to be overlapping. As well as checking for overlaps, the dimer calculations code checks if each dimer is catenated by checking the volume of the pore of the cage alone and comparing that to the volume of the pores within the dimer using PoreMapper `https://github.com/andrewtarzia/PoreMapper`. Cages are considered catenated if the pores in the dimer were smaller than for a single cage, where the threshold was set to 0.1Å^3 to allow for some discrepancies between the two calculations.

12 Pore size distribution

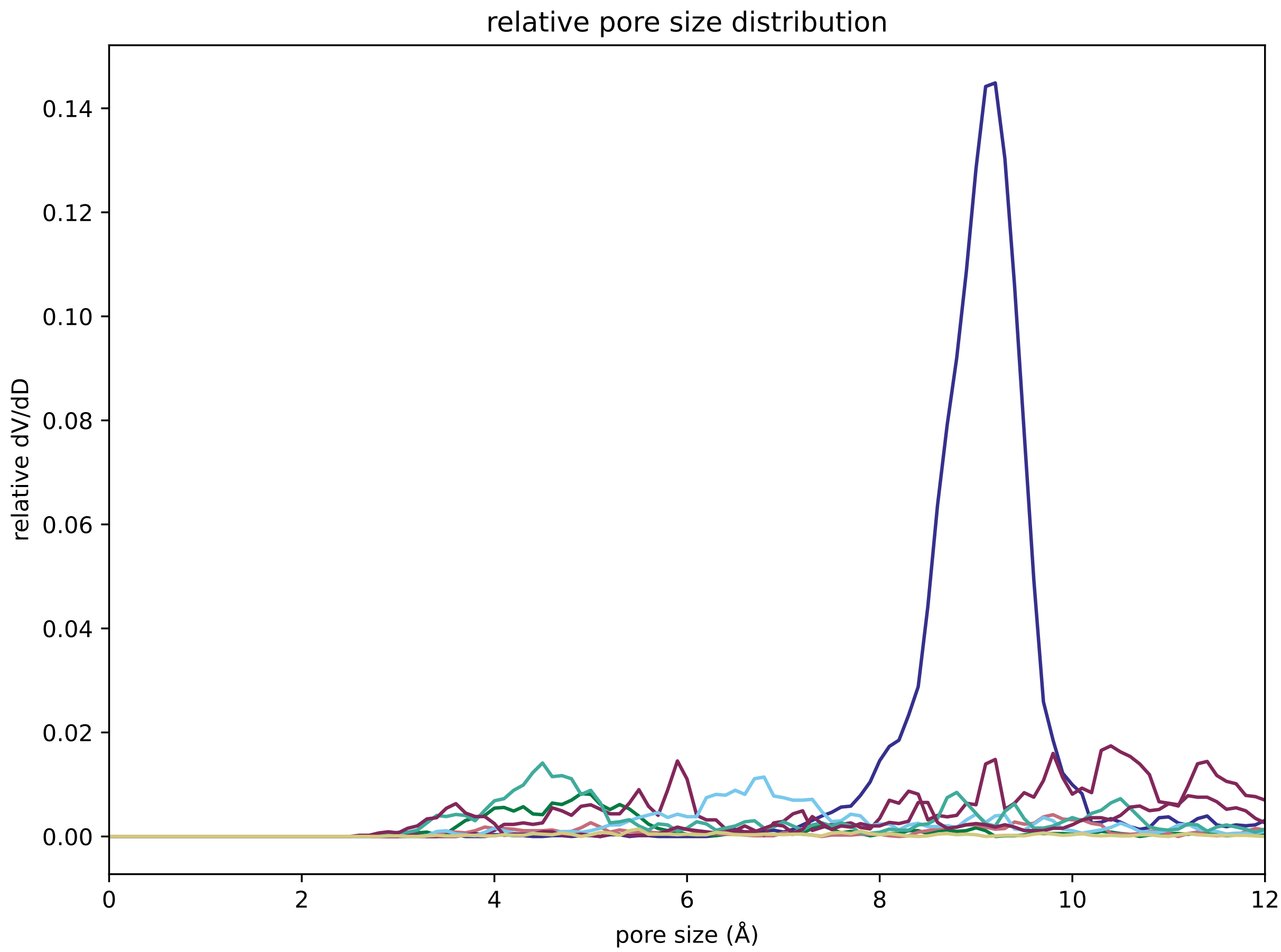

Figure S9: Pore size distribution for the different structures produced in the simulations. The colour of the plot relates to the phase it represents as coloured in Fig. 6. The derivative distribution for each structure is divided by the total void volume of the structure. Source data are provided as a Source Data file.

References

(1) Landrum, G.; Tosco, P.; Kelley, B.; Rodriguez, R.; Cosgrove, D.; Vianello, R.; sriniker; Gedeck, P.; Jones, G.; Kawashima, E.; NadineSchneider; Nealschneider, D.; Dalke, A.; Swain, M.; Cole, B.; tadhurst cdd; Turk, S.; Savelev, A.; Vaucher, A.; Wójcikowski, M.; Take, I.; Walker, R.; Scalfani, V. F.; Faara, H.; Ujihara, K.; Probst, D.; Maeder, N.; Monat, J.; Lehtivarjo, J.; godin, g. rdkit/rdkit: 2025_03_3 (Q1 2025) Release. 2025; https://zenodo.org/records/15605628.
