# Supplementary material for: The role of shape and interaction directionality in the crystalline phase behaviour of octahedral metal-organic cages
Source: Nat Commun. 2026 Sep 22;17:11044. doi: 10.1038/s41467-026-77857-7 (PMC13690734; doi:10.1038/s41467-026-77857-7)
Supplement: Supplementary file 2 [file 41467_2026_77857_MOESM3_ESM.pdf]

nature portfolio

Peer Review File

The role of shape and interaction directionality in the crystalline phase behaviour of octahedral metal-organic cages

Corresponding Author: Dr Emma Wolpert

This file contains all reviewer reports in order by version, followed by all author rebuttals in order by version.

Version 0:

Reviewer comments:

Reviewer #1

(Remarks to the Author)
In this manuscript, Wolpert et al. report an efficient computational approach for predicting the packing structure of octahedral metal-organic cages (MOCs), which is closely relevant for their solid-state material properties. This approach is divided into two main steps: (1) semi-empirical, atomistic dimer-model calculations to identify the dominant intermolecular interaction mode, followed by (2) coarse-grained Monte Carlo simulations of MOC assembly using the polyhedral model with patchy interaction sites on its surface. By modeling the MOC molecules with simpler polyhedra that have variable truncation and interaction directionality, key geometric factors influencing phase selection can be analyzed, and this approach greatly reduces computational expenses. The manuscript is well-described with the details of each procedure, and the results are properly discussed not only based on the geometrical reasonings but also from the viewpoint of the MOC chemistry. However, the generalizability and limitations of the proposed approach are not fully clear, limiting the impact of this manuscript to a wider community. I believe this manuscript meets the criteria of publication quality in Nature Communications by addressing the following points:
(1) As stated in the first sentence of the abstract, the main issue that this computational approach overcame is the high expense of simulating the assembly of large MOC molecules. However, quantitative comparisons from the conventional CSP method or calculations on the smaller POC systems with a similar method (reference [24] and [25]) are not provided. I suggest that the authors give more detailed numerical comparisons on the system size or computational wall times, clarifying the effectiveness of this method. Visual comparisons of the target molecules from different works, such as T2 (A. I. Cooper and G. M. Day et al., Nature 2017, 543, 657–664.), POCs, and the MOC would also help the readers to understand the difference between these systems.
(2) In line 24 on page 2, the manuscript says “The geometry of the cage is primarily governed by the coordination preferences of the metal ions and flexibility of the ligands, but…[7]”. This statement might be misleading for some readers because higher flexibility of the ligand allows a greater variety of cage geometries to be formed, rather than targeting a specific cage geometry. The corresponding reference [7] also highlights such an example. Similar analysis can also be found in the meta-analysis of MOCs (D. Fujita and S. Furukawa, Coord. Chem. Rev. 2022, 467, 214612.), and the ligand coordination angle (= ligand molecular geometry) is described to be an important factor for defining the cage geometry. Thus, to avoid any confusion, the corresponding sentence in the manuscript should be rephrased like: “The geometry of the cage is primarily governed by the coordination preferences of the metal ions and geometric character of the ligands”.
(3) The Figure 1 caption says “X is a H atom”, but X is defined as a corresponding amino-acid code in the original reference [15] (like “Phe” for phenylalanine). If the authors intended to mean that hydrogen atoms are located on the linker in this figure, the corresponding amino acid is glycine, thus X = Gly.
(4) The semi-empirical GFN-xTB method is indeed parameterized to account for non-covalent interactions, but it is worth confirming if this method is accurate enough for the quantitative discussion provided on page 8. I suggest that the authors provide the absolute interaction energy for the facet-to-facet dimer in vacuum, so that this value can be compared with the reported result calculated with the higher levels of theory (LRC-ωPBE/6-311G+MBD-correction; S. Tokuda and S. Furukawa, Nat. Chem. 2025, 17, 672–678.).
(5) Line 105 on page 6 raises one of the aims of this work as “(2) show that our method can account for solvent effects”. However, solvent-induced pseudo-polymorphism of WaaF-1 is not addressed in the manuscript because of the lack of explicit modelling of DMA molecules, and line 131 of page 8 mentions this limitation as “which is currently beyond the scope of our method”. The authors indeed compared the pair potentials of MOCs in the presence and absence of implicit ethanol

solvent, but it resulted in a similar trend of relative energies, and the further discussions on the solvent effect are unfortunately not provided in the following paragraphs. Thus, unless more detailed discussions are provided, the authors should avoid stating that this approach can address the solvent effect. For a possible discussion, I would suggest additionally calculating the dimer energies under the vacuum/unconstrained/normal condition so that one can assess the effect of implicit solvent more reliably. If one can see that the facet-to-facet interaction mode is more preferred to the other interaction modes in the presence of an implicit solvent, it is possible to suggest the importance of solvents for the selective formation of the WaaF-1 phase.
(6) Following the last comment (5), I suggest plotting the relative energies in Table 1 in a bar plot to facilitate the comparisons among the data sets.
(7) Line 222 on page 13 describes that the temperature for Monte Carlo (MC) simulation is reduced according to T(i+1) = T(i)*0.95, but this symbol is not defined in the manuscript nor supplementary sections. The authors should provide the definition of T(i) or should describe this method in different words.
(8) As described in line 234 on page 13, the final configuration of MC simulation was evaluated based on the radial distribution functions (RDFs) that are cut-offed at 1.9 Å as the maximum. This means the packing order is inspected as a local cluster containing a few layers of polyhedra around the centre. However, “crystal structure” is, by definition, based on a long-range order spanning the space, conflicting with this evaluation method. Even if such long-range order was visually confirmed in this study, it would be a recommendable practice to show what a simulation box looks like after forming the “crystalline” phases to prove the validity of this evaluation method. I suggest that the authors provide at least one figure representing a whole simulation box after the MC cycles, either in the main manuscript or supporting material, like the one in the reference [24]. Such a figure would also facilitate the readers' understanding of this computational approach.
(9) Figure 6 is aesthetically pleasing, but not colour-blind friendly. I recommend defining a symbol or label for each packing phase and putting them in the phase diagram (a) and the RDF plot (e).
(10) Figure 7 is difficult to interpret for several reasons; (1) there is no explanation for the schematic illustrations in the column title (packing of polyhedra?). Please provide proper descriptions in the caption or explain them in the main text. (2) The green-yellow-red colour code is not intuitive to understand the order and is not colour-blind friendly. I suggest using a triangular radar chart to represent the trade-off relations of the three factors. Ideally, these three factors (interparticle distance, facet-to-facet contact, and coordination number) should be quantified for each packing phase from the corresponding RDFs and contact area data (Figure S1), and this information can be depicted in a triangular radar chart. Such a diagram would greatly make the trade-off picture more convincing.
(11) In line 365 on page 19, a white space is missing in “0.1Å” between the number and unit.
(12) In line 462 on page 23, a close parenthesis is missing in “[Rh12(NDI-Phe)12(pyridine6]”.
(13) As the dimer calculations are also performed in the implicit solvent model, line 488 on page 25 “the packing behaviour of desolvated, neutral MOCs” should be modified to “the packing behaviour of charge-neutral MOCs”.

(Remarks on code availability)

Reviewer #2

(Remarks to the Author)
The authors report a proof-of-concept of predicting phases of metal-organic cages (MOCs) using course-grained molecular simulations. The approach is an elegant combination of applied mathematics where the geometry is approximated with polyhedra, and molecular simulation where interactions and statistical configurations are incorporated. The authors compare their results with two experimental MOCs, showing reasonably good agreement. With the recent Nobel prize for MOFs, there is a renewed interest in these structures, and MOCs are a similar analogue to MOFs with possibly more variations given the multiple phases that can occur.

I recommend to publish with minor adjustments based on the comments below.

1. Equation 1 shows a multiplication of potential energies from LJ, angular and torsional terms. However, I would have thought these energies should be additive. Please comment.
2. I am interested in the solvent effects explored using the ALPB model for the high symmetry caes. There was a recent paper showing that the dielectric constant controls the phases of resilin molecules (DOI: 10.1126/sciadv.abq2202). I'd like to authors to explain their work further. The ALPB model requires partial charges and dielectric parameters, however none of these details are provided. Were the charges placed at the center of each face? How were the partial charges determined?
3. The observation of grain boundaries are interesting. Can the authors present some figures showing this? I'm trying to visualise where the grain boundaries are formed? In a plane across a crystal, in a radial configuration, or otherwise?
4. Very impressed to see a phase diagram in Figure 6, with very clear color coding. Nice work. I wonder whether this diagram could include some points that represent experiment structures. No need to generate another figure, but interested to hear whether the authors can commment.
5. I understand the model has used a face with the center of the face as the interaction point. Have the authors considered placing LJ interaction points on the cornices or sides of a face? I imagine this could more accuratly model MOCs with strongly interacting groups at the corners.
6. I'm not sure what a lithium phase is? page 16. What's the relevance? Because it is a well known material of interest?
7. Degree of crystallisation. I imagine that not all the cages aggregate together into a phase. This may also be a good metric to consider using. Degree of crystallisation, number of cages in a phase divided by all cages in the cell?
8. Did the authors try adjusting the LJ potential well depth parameter? Or did they just use off-the=shelf forcefield? It would

be interesting to play with this parameter and see shifts in phases.

(Remarks on code availability)
I didn't attempt to run the code, but I can view and read the intructions. It looks fairly startighforward.

Reviewer #3

(Remarks to the Author)
This article on predicting the sold-state structures of MOCs (i.e., packing behavior) based on inexpensive computations is extremely well presented and provides a valuable contribution to the literature. The phase diagrams, in particular, are a highlight of this work. My only minor critique is that Figure 7 could benefit from the criteria for being most/least favoured being more clear in the Figure (or caption). Otherwise I recommend publishing the paper as is.

(Remarks on code availability)

Reviewer #4

(Remarks to the Author)
I co-reviewed this manuscript with one of the reviewers who provided the listed reports. This is part of the Nature Communications initiative to facilitate training in peer review and to provide appropriate recognition for Early Career Researchers who co-review manuscripts.

(Remarks on code availability)

Version 1:

Reviewer comments:

Reviewer #1

(Remarks to the Author)
The authors addressed all the points raised by the reviewers and improved the manuscript accordingly. I believe that the current version of the manuscript meets the publication criteria of Nature Communications and is ready to be published as is.

Shun Tokuda

(Remarks on code availability)

Reviewer #2

(Remarks to the Author)
The authors have adequately addressed my comments. I recommend publication of this manuscript.

(Remarks on code availability)

Open Access This Peer Review File is licensed under a Creative Commons Attribution 4.0 International License, which permits use, sharing, adaptation, distribution and reproduction in any medium or format, as long as you give appropriate credit to the original author(s) and the source, provide a link to the Creative Commons license, and indicate if changes were made.
In cases where reviewers are anonymous, credit should be given to 'Anonymous Referee' and the source.
The images or other third party material in this Peer Review File are included in the article's Creative Commons license, unless indicated otherwise in a credit line to the material. If material is not included in the article's Creative Commons license and your intended use is not permitted by statutory regulation or exceeds the permitted use, you will need to obtain permission directly from the copyright holder.
To view a copy of this license, visit https://creativecommons.org/licenses/by/4.0/

The authors thank the reviewers for their thoughtful suggestions and below we respond to the comments point-by-point. The reviewers' comments are given in black font, our response in blue and changes in the manuscript are highlighted in blue.

REVIEWER COMMENTS

Reviewer #1 (Remarks to the Author):

In this manuscript, Wolpert et al. report an efficient computational approach for predicting the packing structure of octahedral metal-organic cages (MOCs), which is closely relevant for their solid-state material properties. This approach is divided into two main steps: (1) semi-empirical, atomistic dimer-model calculations to identify the dominant intermolecular interaction mode, followed by (2) coarse-grained Monte Carlo simulations of MOC assembly using the polyhedral model with patchy interaction sites on its surface. By modeling the MOC molecules with simpler polyhedra that have variable truncation and interaction directionality, key geometric factors influencing phase selection can be analyzed, and this approach greatly reduces computational expenses. The manuscript is well-described with the details of each procedure, and the results are properly discussed not only based on the geometrical reasonings but also from the viewpoint of the MOC chemistry. However, the generalizability and limitations of the proposed approach are not fully clear, limiting the impact of this manuscript to a wider community. I believe this manuscript meets the criteria of publication quality in Nature Communications by addressing the following points:

(1) As stated in the first sentence of the abstract, the main issue that this computational approach overcame is the high expense of simulating the assembly of large MOC molecules. However, quantitative comparisons from the conventional CSP method or calculations on the smaller POC systems with a similar method (reference [24] and [25]) are not provided. I suggest that the authors give more detailed numerical comparisons on the system size or computational wall times, clarifying the effectiveness of this method. Visual comparisons of the target molecules from different works, such as T2 (A. I. Cooper and G. M. Day et al., Nature 2017, 543, 657–664.), POCs, and the MOC would also help the readers to understand the difference between these systems.

We thank the reviewer for their suggestion. We agree that adding numerical context for the computational cost makes the advantage of the approach clearer. We had not originally included a direct comparison because the computational costs are difficult to compare across methods and systems. For example, the reported crystal structure prediction cost for T2E from Pulido *et al.* involves a different molecular system, different flexibility, different atom count, different solvent treatment, and different levels of theory for parametrisation. Although CSP has been applied to porous organic cages, and recent work has used CSP workflows to generate representative solid-state structures for selected metal-organic polyhedra, we are not aware of an example where conventional CSP has been used systematically to predict the crystal structure landscape of MOCs. Direct comparison with purely organic systems is imperfect as the inclusion of metal centres introduces additional challenges for molecular parametrisation and crystal-energy ranking.

Nevertheless, we agree that an approximate comparison is useful. We have therefore added a section to the SI estimating the CPU-hours required for both the dimer calculations and the HPMC simulations in this work, and comparing these values with the reported cost for T2E. We also clarify that these values are not fully optimised: the dimer calculations were run as a proof-of-concept and could be made more efficient, and the HPMC simulations could be accelerated by using an adaptive cooling schedule. For example, adapting the cooling rate based on the heat capacity to cool slowly over the phase transition but quickly away from the transition temperature. We have not added a visual comparison to T2E in the manuscript because we feel it would distract from the focus of the paper. Instead, we report on the number of atoms in the two different molecules to highlight the differences.

"To estimate the computational cost required for structure prediction of a new MOC using our workflow, we calculated the CPU-hours required for both the dimer calculations and HPMC simulations. We note that these values should be regarded as an upper estimate for the present proof-of-concept implementation as the cost of dimer calculations could be reduced by optimising the sampling procedure, and the cost of the HPMC simulations could be reduced by employing an adaptive cooling rate. For example, the cooling rate could be adjusted based on the heat capacity, allowing the system to cool slowly through the phase the phase transition but more rapidly away from the transition temperature" [54]

The total cost of the dimer calculations used to identify the low energy implicitly solvated dimers across the different constrained/unconstrained and optimisation conditions was 62,117 CPU-hours. For the HPMC simulations, each simulation run required 24 hours using 64 cores, corresponding to 1536 CPU-hours. Therefore, the approximate computational cost for predicting one crystal structure using a single HPMC simulation is 64,000 CPU-hours. More conservatively, accounting for multiple simulations across a range of input parameters and repeated runs for each parameter set, the total cost may be closer to 100,000 CPU-hours.

For context, Pulido et al.[55] reported that determining the crystal energy landscape of T2E, an extended triptycene trisbenzimidazolone analogue, took 400,000 CPU-hours. A direct comparison between the two studies is necessarily approximate, since the systems differ in size, flexibility, chemical composition, solvent environment, and level of theory/parameterisation. In particular, the system studied here is a MOC with 822 atoms, whereas T2E is a purely organic molecule containing 64 atoms. Nevertheless, this comparison indicates that the dimer-informed coarse-grained workflow can provide access to crystal packing predictions for larger and more complex cage-like systems at a reduced computational cost relative to conventional CSP studies."

(2) In line 24 on page 2, the manuscript says "The geometry of the cage is primarily governed by the coordination preferences of the metal ions and flexibility of the ligands, but…[7]". This statement might be misleading for some readers because higher flexibility of the ligand allows a greater variety of cage geometries to be formed, rather than targeting a specific cage geometry. The corresponding reference [7] also highlights such an example. Similar analysis can also be found in the meta-analysis of MOCs (D. Fujita and S. Furukawa, Coord. Chem. Rev. 2022, 467, 214612.), and the ligand coordination angle (= ligand molecular geometry) is described to be an important factor for defining the cage geometry. Thus, to avoid any confusion, the corresponding sentence in the manuscript should be rephrased like: "The geometry of the cage is primarily governed by the coordination preferences of the metal ions and geometric character of the ligands".

We have changed the sentence in the manuscript to the following: "The geometry of the cage is primarily governed by the coordination preferences of the metal ions and geometry of the ligands".

(3) The Figure 1 caption says "X is a H atom", but X is defined as a corresponding amino-acid code in the original reference [15] (like "Phe" for phenylalanine). If the authors intended to mean that hydrogen atoms are located on the linker in this figure, the corresponding amino acid is glycine, thus X = Gly.

We thank the author for pointing out our mistake here and have changed the figure caption accordingly.

(4) The semi-empirical GFN-xTB method is indeed parameterized to account for non-covalent interactions, but it is worth confirming if this method is accurate enough for the quantitative discussion provided on page 8. I suggest that the authors provide the absolute interaction energy for the facet-to-facet dimer in vacuum, so that this value can be compared with the reported result calculated with the higher levels of theory (LRC-ωPBE/6-311G+MBD-correction; S. Tokuda and S. Furukawa, Nat. Chem. 2025, 17, 672–678.).

We agree that, while GFN2-xTB is parameterised to account for non-covalent interactions, the quantitative accuracy of the interaction energies should be assessed. We have therefore calculated the interaction energy for the lowest-energy facet-to-facet dimer in vacuum. Our calculation gives and interaction energy of -350 kJ/mol compared to the reported value by Tokuda and Furukawa of -433 kJ/mol. The GFN2-xTB value is therefore smaller in magnitude by around 80 kJ/mol, but captures the same qualitative picture of a very strongly stabilised facet-to-facet dimer. We have added the following to Section 4.1:

"To assess the quantitative accuracy of the GFN2-xTB interaction energies, we calculated the absolute interaction energy of the lowest-energy dimer in vacuum. Geometry optimisations were performed on dimers in the gas phase using the optimisation levels `crude', `sloppy', `lax', and `normal', where dimers within the lowest 100 kJ mol^{-1} were taken forward for calculations at stricter optimisation levels, as well as the lowest-energy dimer for each packing type if none were found within 100 kJ mol^{-1}. We then performed a final optimisation, removing the constraints between metal-metal ions, confirming the lowest energy dimer was facet-to-facet with an interaction energy of -350 kJ mol^{-1}. Comparing this to the -433 kJ mol^{-1} reported by Tokuda and Furukawa[15] using LRC- ωPBE/6-311G with MBD correction shows that although GFN2-xTB underestimates the magnitude of the interaction by approximately 80 kJ mol^{-1} it captures the same qualitative conclusion that the facet-to-facet dimer is strongly stabilised."

The dimer calculations are used primarily to identify the dominant packing motif and relative energetic ordering of candidate motifs. Because our argument on page 8 relies primarily on the relative energetic ordering rather than absolute energy, the comparison with the higher-level calculation supports the qualitative conclusion that the facet-to-facet motif is strongly stabilised. While we cannot assume that the GFN2-xTB error is strictly systematic across all structures, the same order of magnitude for the facet-to-facet interaction and the large difference in energy between facet-to-facet and the next lowest energy interaction, edge-to-edge, indicates the method captures the dominant interaction motif.

(5) Line 105 on page 6 raises one of the aims of this work as "(2) show that our method can account for solvent effects". However, solvent-induced pseudo-polymorphism of WaaF-1 is not addressed in the manuscript because of the lack of explicit modelling of DMA molecules, and line 131 of page 8 mentions this limitation as "which is currently beyond the scope of our method". The authors indeed compared the pair potentials of MOCs in the

presence and absence of implicit ethanol solvent, but it resulted in a similar trend of relative energies, and the further discussions on the solvent effect are unfortunately not provided in the following paragraphs. Thus, unless more detailed discussions are provided, the authors should avoid stating that this approach can address the solvent effect. For a possible discussion, I would suggest additionally calculating the dimer energies under the vacuum/unconstrained/normal condition so that one can assess the effect of implicit solvent more reliably. If one can see that the facet-to-facet interaction mode is more preferred to the other interaction modes in the presence of an implicit solvent, it is possible to suggest the importance of solvents for the selective formation of the WaaF-1 phase.

We thank the reviewer for their comment. We agree that the original wording could have been read as implying we could reproduce the full solvent-induced pseudopolymorphism as previously reported. This was not our intention as the DMA-stabilised distorted FCC phase likely requires explicit modelling of DMA which is beyond the scope of the present work. Our aim was instead to test whether implicit solvent could be incorporated into the dimer-informed workflow and whether, under implicit solvent conditions, the method predicts the experimentally observed packing behaviour. The GFN2-xTB/ALPB calculations do predict the facet-to-facet dimer as the preferred motif, which then maps onto the experimental crystal structure.

Following the reviewer's suggestion we also performed dimer calculations under vacuum. These show the same qualitative ordering as the implicit ethanol calculations. We therefore do not claim that ethanol selectively stabilises WaaF-1, rather that including implicit solvent in the dimer calculations is compatible with predicting the experimentally observed phase. We have included the vacuum/unconstrained/normal results and changed the following in the manuscript:

Our second aim is now: "test whether an implicit solvent dimer-informed workflow can predict the packing observed experimentally"

The discussion on page 9 includes: "We also performed the same optimisation protocol under vacuum to assess the sensitivity of this result to implicit-solvent treatment. These calculations showed the same qualitative energetic ordering as the implicit-ethanol calculations, with the facet-to-facet motif remaining the lowest-energy arrangement. We therefore do not interpret the GFN2-xTB/ALPB calculations as showing that ethanol selectively stabilises the WaaF-1 phase relative to vacuum. Instead, they show that when ethanol is included through an implicit-solvent model, facet-to-facet packing remains preferred, consistent with the experimentally observed packing motif."

And in the conclusion: "The dimer calculations, performed with implicit solvation, captured the observed facet-to-facet packing behaviour, which allowed the experimentally reported WaaF-1 structure to be recovered in the coarse-grained simulations. The equivalent vacuum calculations show the same qualitative energetic ordering, indicating that this packing preference is robust rather than uniquely stabilised by an implicit representation of ethanol. Therefore, while the present calculations do not address the full solvent-induced pseudopolymorphism reported experimentally, which would require explicit treatment of DMA and specific solvent-cage interactions, they show that implicit solvation can be incorporated into the dimer-informed workflow while retaining the ability to predict the experimentally observed WaaF-1 packing motif. Combined with the efficiency of the HPMC simulations, this workflow offers a realistic route to high-throughput screening of MOC phase space to identify candidates with desirable properties, such as high porosity."

(6) Following the last comment (5), I suggest plotting the relative energies in Table 1 in a bar plot to facilitate the comparisons among the data sets.

We thank the reviewer for their suggestion that makes the data easier to understand. We have replaced the table with Figure 4 (see below) and moved the table to the SI.

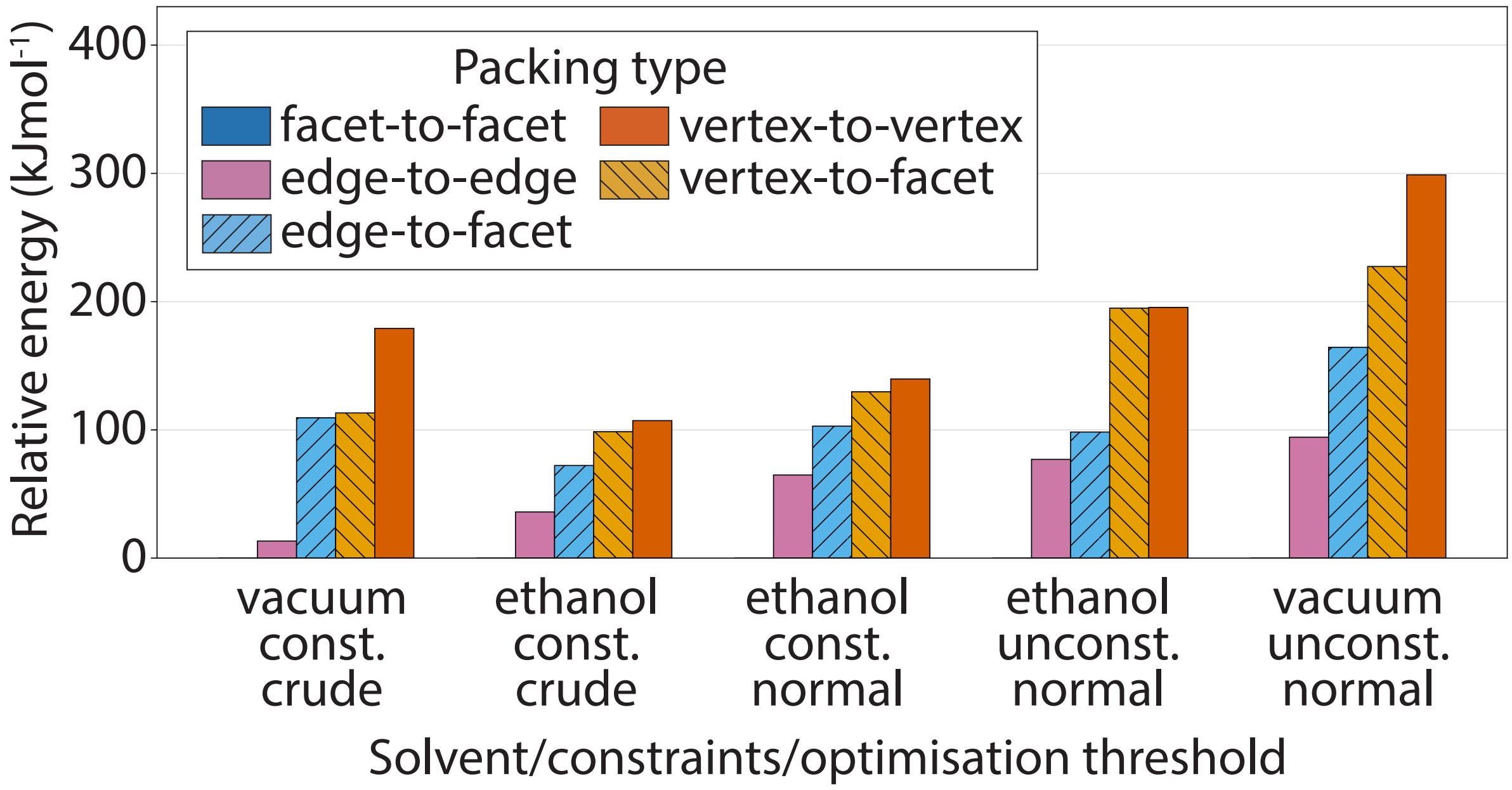

(7) Line 222 on page 13 describes that the temperature for Monte Carlo (MC) simulation is reduced according to T(i+1) = T(i)*0.95, but this symbol is not defined in the manuscript nor supplementary sections. The authors should provide the definition of T(i) or should describe this method in different words.

We have added the following to the sentence: "The temperature at each step, T(i), was reduced according to…"

(8) As described in line 234 on page 13, the final configuration of MC simulation was evaluated based on the radial distribution functions (RDFs) that are cut-offed at 1.9 Å as the maximum. This means the packing order is inspected as a local cluster containing a few layers of polyhedra around the centre. However, "crystal structure" is, by definition, based on a long-range order spanning the space, conflicting with this evaluation method. Even if such long-range order was visually confirmed in this study, it would be a recommendable practice to show what a simulation box looks like after forming the "crystalline" phases to prove the validity of this evaluation method. I suggest that the authors provide at least one figure representing a whole simulation box after the MC cycles, either in the main manuscript or supporting material, like the one in the reference [24]. Such a figure would also facilitate the readers' understanding of this computational approach.

We thank the reviewer for their suggestion and have included a figure with representative clusters of each different phase (see below) in the SI, along with the following sentence in the manuscript: "Example clusters of each of the seven phases as formed in the simulations are given in Figure S3."

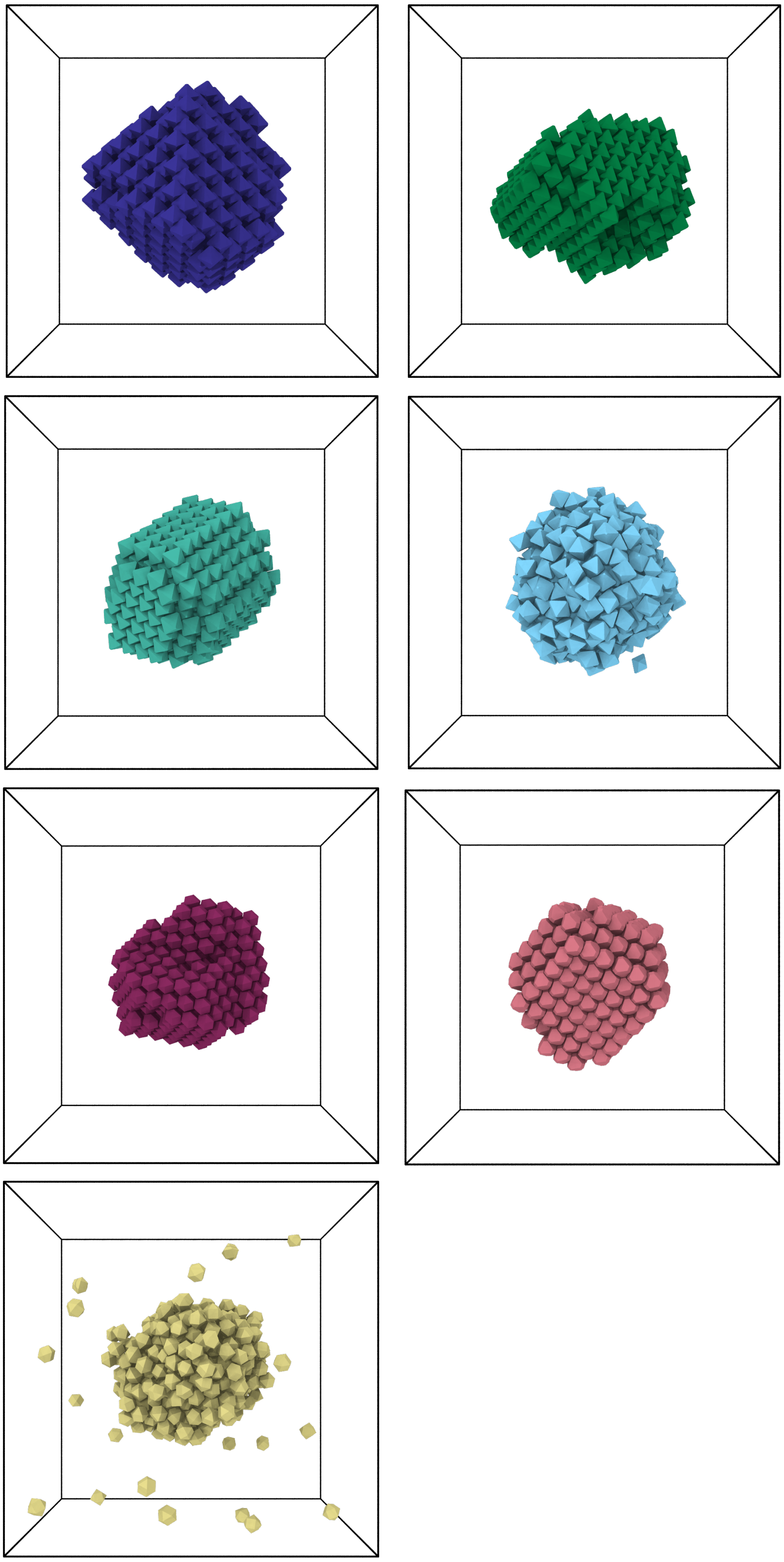

(9) Figure 6 is aesthetically pleasing, but not colour-blind friendly. I recommend defining a symbol or label for each packing phase and putting them in the phase diagram (a) and the RDF plot (e).

We thank the reviewer for their suggestion. The colours in Fig. 6 were chosen from the colour blind friendly Tol pallete from https://davidmathlogic.com/colorblind/. We have therefore retained the colour scheme but have avoided adding additional labels or symbols directly to the phase diagram because this would overcrowd the figure and make the phase boundaries harder to read.

(10) Figure 7 is difficult to interpret for several reasons; (1) there is no explanation for the schematic illustrations in the column title (packing of polyhedra?). Please provide proper descriptions in the caption or explain them in the main text. (2) The green-yellow-red colour code is not intuitive to understand the order and is not colour-blind friendly. I suggest using a triangular radar chart to represent the trade-off relations of the three factors. Ideally, these three factors (interparticle distance, facet-to-facet contact, and coordination number) should be quantified for each packing phase from the corresponding RDFs and contact area data (Figure S1), and this information can be depicted in a triangular radar chart. Such a diagram would greatly make the trade-off picture more convincing.

We thank the reviewer for the helpful suggestion. We have added in a new figure with a radial chart into our analysis (Fig 8(b) and Fig S5) and believe it has improved our analysis of the structures.

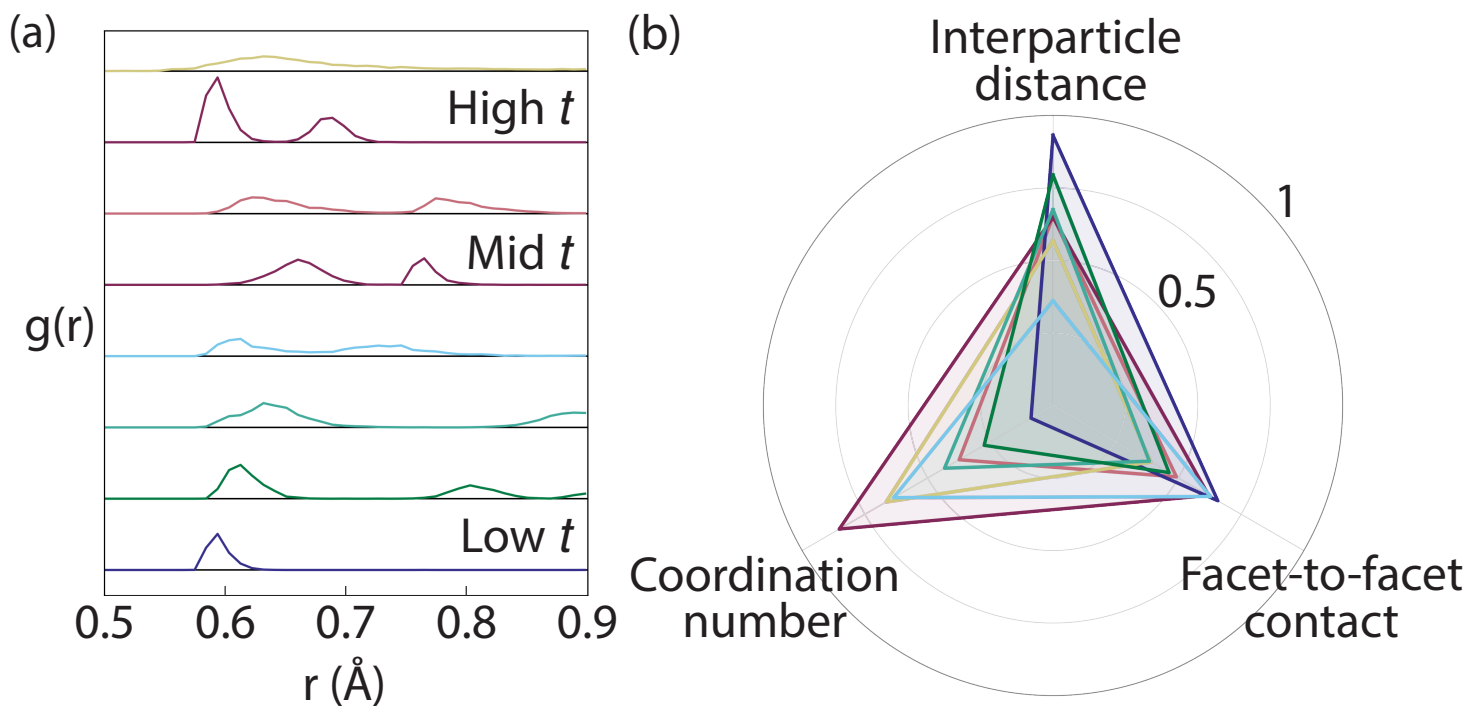

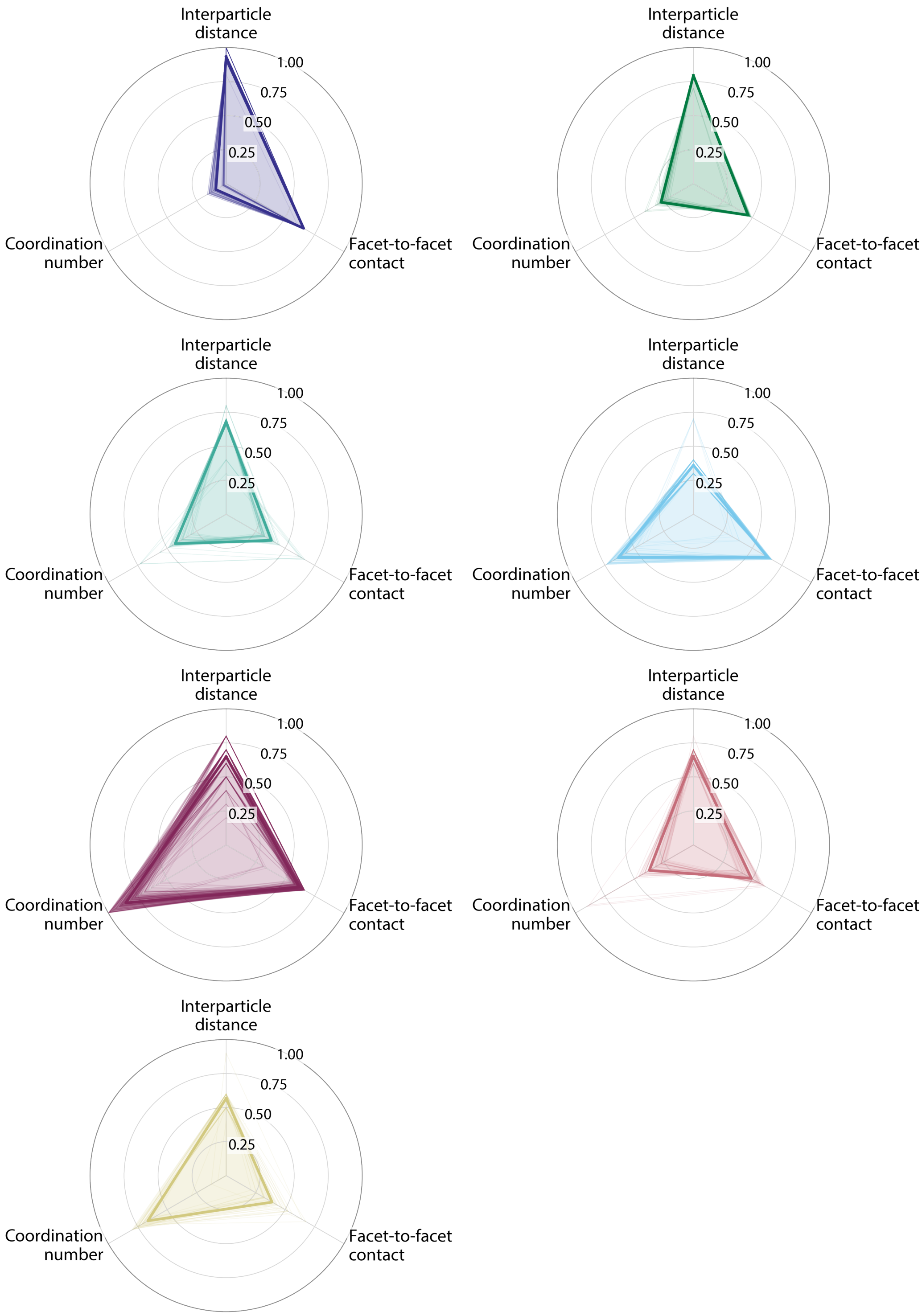

We have also moved Figure 7 to the SI and added a figure title whilst changing the colours to be more colour blind friendly according to the Wong colour palette given on the website https://davidmathlogic.com/colorblind/.

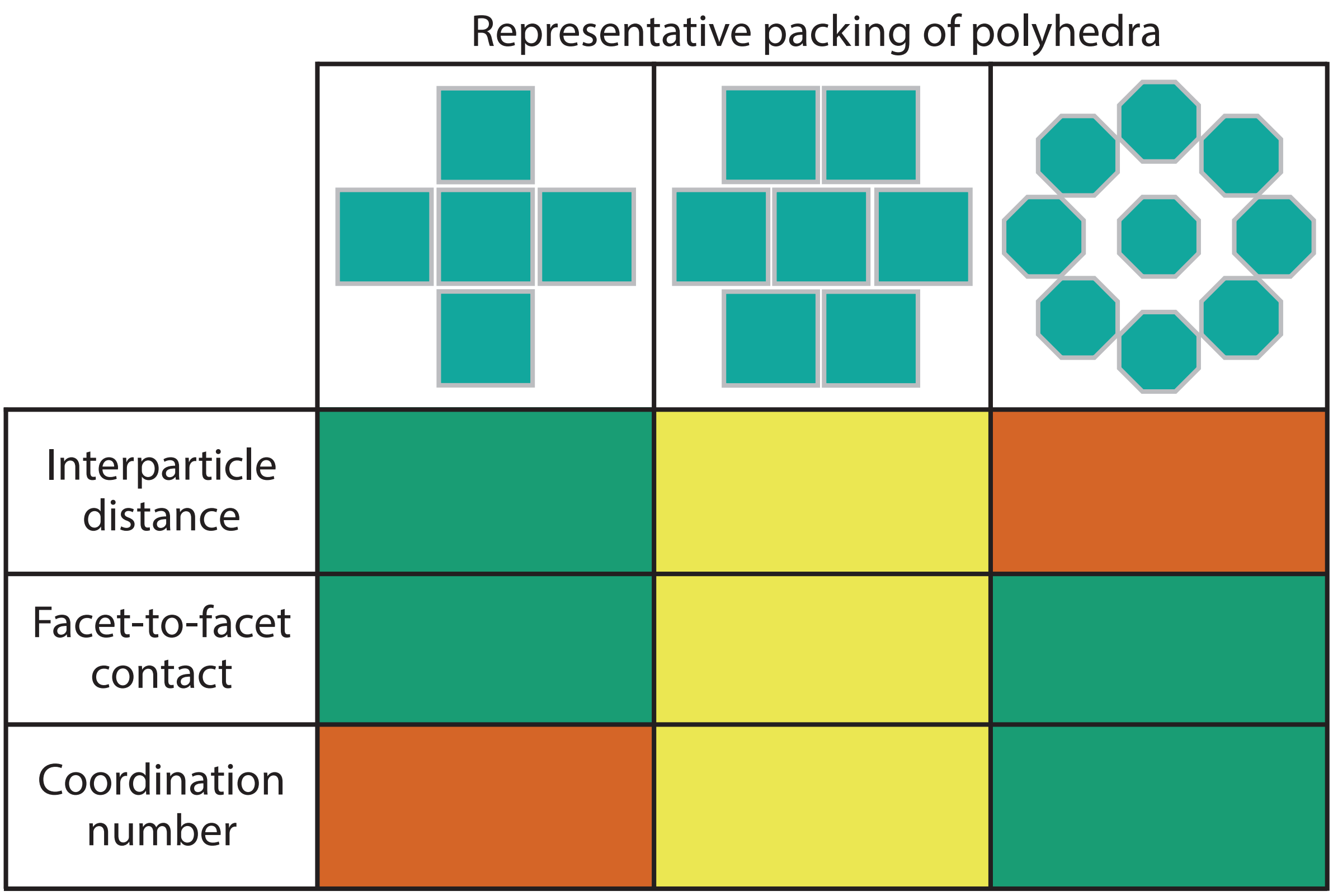

(11) In line 365 on page 19, a white space is missing in “0.1Å” between the number and unit.

We have added the white space.

(12) In line 462 on page 23, a close parenthesis is missing in “[Rh12(NDI-Phe)12(pyridine6]”.

We have added the parenthesis.

(13) As the dimer calculations are also performed in the implicit solvent model, line 488 on page 25 “the packing behaviour of desolvated, neutral MOCs” should be modified to “the packing behaviour of charge-neutral MOCs”.

We thank the reviewer for their suggestion and changed the sentence accordingly.

Reviewer #2 (Remarks to the Author):

The authors report a proof-of-concept of predicting phases of metal-organic cages (MOCs) using course-grained molecular simulations. The approach is an elegant combination of applied mathematics where the geometry is approximated with polyhedra, and molecular simulation where interactions and statistical configurations are incorporated. The authors compare their results with two experimental MOCs, showing reasonably good agreement. With the recent Nobel prize for MOFs, there is a renewed interest in these structures, and MOCs are a similar analogue to MOFs with possibly more variations given the multiple phases that can occur.

I recommend to publish with minor adjustments based on the comments below.

1. Equation 1 shows a multiplication of potential energies from LJ, angular and torsional terms. However, I would have thought these energies should be additive. Please comment.

The reviewer is correct that independent energetic contributions are commonly additive, but in this case the angular and torsional terms are not separate energies but act as modulators which give directionality to the interaction by switching the radial attraction on and off depending on the relative orientation of the two particles. This ensures that the attractive interaction only occurs when the particles are both at a correct separation and correctly oriented. If the terms were additive then a favourable LJ contact could still contribute substantial attraction even when the molecules are badly misaligned, which would not be recreating the behaviour of the intermolecular interactions. We have clarified this in the manuscript under equation 1: “Here, V_{ang} and V_{tor} are dimensionless orientational modulation terms rather than independent energetic contributions, such that they

switch the Lennard-Jones attraction on or off depending on the relative angular and torsional alignment of the patches." As well as editing our description of V_{ang} and V_{tor}: "V_{ang} and V_{tor} modulate the Lennard-Jones potential by reducing the magnitude of the attractive interaction for configurations that that misalign the patches (Vang) or introduce rotations away from the preferred alignment (Vtor)."

2. I am interested in the solvent effects explored using the ALPB model for the high symmetry caes. There was a recent paper showing that the dielectric constant controls the phases of resilin molecules (DOI: 10.1126/sciadv.abq2202). I'd like to authors to explain their work further. The ALPB model requires partial charges and dielectric parameters, however none of these details are provided. Were the charges placed at the center of each face? How were the partial charges determined?

We agree that the dielectric sensitivity of molecular phase behaviour is an important broader point, as illustrated by the resilin study cited by the reviewer. However, our use of ALPB is more limited in scope. We use it as an efficient way to compare the energetic trends between candidate cage dimers. The solvent effects reported were not obtained by constructing an external charge model or by placing charges at the centre of each face, but rather by using the ALPB solvation model as implemented in xTB. In this implementation the electrostatic part of the ALPB solvation free energy is evaluated using atom-centred partial charges generated internally by the calculation, together with the dielectric constant/solvent parameters defined for the chosen built-in solvent model. These solvent parameters are taken from the xTB/ALPB parameterisation for ethanol, rather than fitted or modified in this work. To make this clearer we include the following in our methods section in the paper: "The partial charges used by ALPB were those generated internally by the GFN2-xTB calculation for each atomistic structure. The solvent dielectric constant and non-electrostatic ALPB parameters were taken from the built-in xtb/ALPB solvent parameter set for ethanol, corresponding to reference parameters used by the xTB implementation."

3. The observation of grain boundaries are interesting. Can the authors present some figures showing this? I'm trying to visualise where the grain boundaries are formed? In a plane across a crystal, in a radial configuration, or otherwise?

We thank the reviewer for the suggestion. The grain boundaries form in a plane between different orientations of the crystal. We have included a figure in the SI (Fig.S8) of different views of a grain boundary formed in our simulations. As it is hard to view statically, we have also uploaded the ovito session to the ESI for further inspection.

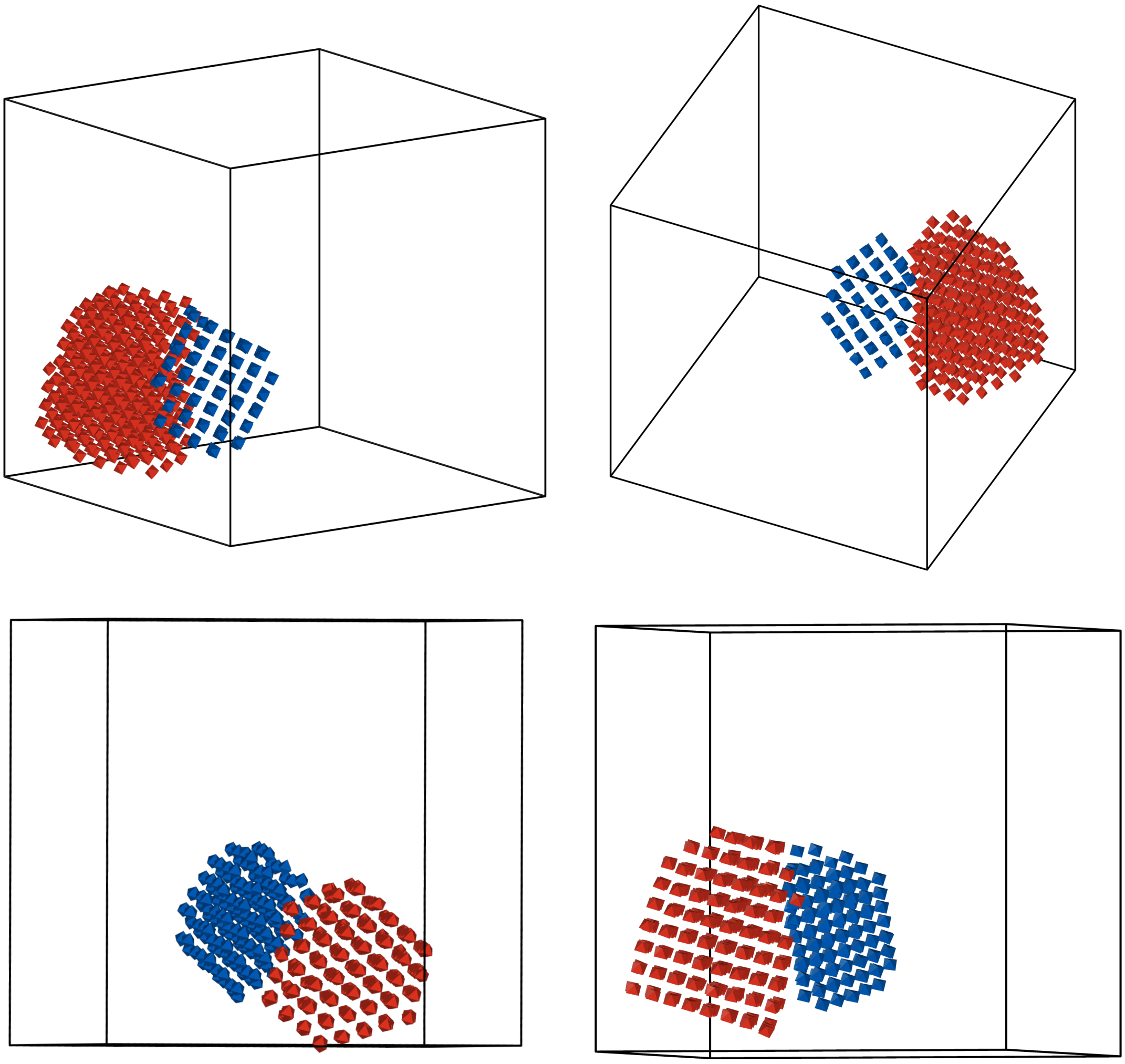

4. Very impressed to see a phase diagram in Figure 6, with very clear color coding. Nice work. I wonder whether this diagram could include some points that represent experiment structures. No need to generate another figure, but interested to hear whether the authors can commment.

We thank the reviewer for the suggestion. This was something we also considered, and we agree that placing experimental structures directly onto the phase diagram would be a useful way to connect the coarse-grained model to real systems. However, at present we think this would risk implying a more quantitative mapping than is currently justified. The truncation parameter can be estimated directly from the cage geometry, and therefore we can position experimental structures along the y-axis, but σ_{ang} is an effective coarse-grained parameter and not a directly measurable molecular quantity. Although the dimer calculations show the MOC in Figure 2a has a strong energetic preference for direct facet-to-facet alignment, and therefore should be represented by the low σ_{ang} region of the phase diagram, we cannot yet assign a precise numerical value of σ_{ang} from dimer calculations alone. Moreover, we have not performed dimer calculations on the other MOC which matches to our phase diagram, so would not be able to place that either.

We have therefore avoided plotting experimental points on the phase diagram as doing so would suggest a quantitative correspondence between the dimer energy landscape and σ_{ang} that has not yet been established. Developing a quantitative mapping between atomistic dimer energy landscapes and σ_{ang} would require a larger set of experimental cage structures spanning multiple regions of the phase diagram, and is an important direction for future work. We have included the following in the paper in Section 2.4 to make this clear: "Whilst the dimer calculations can indicate whether σ_{ang} is qualitatively low or high, we cannot yet assign an exact value because σ_{ang} remains an effective coarse-grained parameter whose quantitative mapping to atomistic dimer energy barriers has not yet been established."

5. I understand the model has used a face with the center of the face as the interaction point. Have the authors

considered placing LJ interaction points on the cornices or sides of a face? I imagine this could more accuratly model MOCs with strongly interacting groups at the corners.

We thank the reviewer for their suggestion. In the present work, we did not include interaction sites on the edges or vertices because the semi-empirical dimer calculations for the MOC studied did not indicate these were dominant interactions. However, we agree that moving the placement of the patches is an important extension of our work as many MOCs have strongly interacting groups located near edges or vertices. This is a direction we plan to explore in future work.

6. I'm not sure what a lithium phase is? page 16. What's the relevance? Because it is a well known material of interest?

We thank the reviewer for pointing out that the phrase "lithium phase" was unclear. We intended to showcase this as a structural comparison. Highlighting that structures in our simulations have been found elsewhere, in this case in high pressure examples of elemental Li. We have edited the text accordingly: "Finally, the phase at highest σ_{ang} light blue in Fig.7(a), contains two sublattices where there are facet-to-facet contacts only within each sublattice, but less directional interactions between the two sublattices (Fig.7(biv)). This gives the same type of crystal connectivity reported to form in elemental lithium under high pressure.[37] This phase is not the Minkowski lattice as formed from simulations of hard octahedra in Ref.34, but is closely related to the BCC phase formed with densely packed octahedra, but with columns of atoms shifted. This structure forms over the denser Minkowski lattice due to the attractive nature of the octahedral facets, and this has also been observed for attractive octahedral nanocrystals.[39]" As well as in the figure caption: "For the light blue phase, the orientations correspond to the view along the [111] (left) and [100] (right) vector in the geometrically equivalent structure formed by lithium under high pressure as reported by Hanfland et al"

7. Degree of crystallisation. I imagine that not all the cages aggregate together into a phase. This may also be a good metric to consider using. Degree of crystallisation, number of cages in a phase divided by all cages in the cell?

We thank the reviewer for this suggestion. In the majority of our simulations, almost all the cages aggregate together into a single cluster, with at most one or two free particles, although grain boundaries sometimes form within the cluster. The behaviour can be seen in the representative cluster figure now included in the SI---see reviewer 1 point 8---(Fig.S3). This is likely because our simulations are cooled very slowly and well beyond the transition temperature such that all particles/cages aggregate together by the final frame. The main exceptions occur at high $\sigma_{ang,}$ for example in the yellow phase in Figure S3. As a result, a degree of crystallisation metric would be close to 1 for most final configurations which is where our analysis is focused and would therefore provide limited additional information. We agree that, were we to study the phase transitions in more detail, this would indeed be a useful metric for tracking the extent of aggregation and crystallisation during assembly. However, the focus of the present study is on the final assembled structures rather than on the transition pathway itself.

8. Did the authors try adjusting the LJ potential well depth parameter? Or did they just use off-the=shelf forcefield? It would be interesting to play with this parameter and see shifts in phases.

This is certainly an interesting suggestion. In this work we did not adjust the LJ potential well depth parameter, as our focus was on the effects of particle shape and interaction directionality. Varying the well depth would be a useful way to explore how the strength of the attractive interaction influences the phase boundaries. This is particularly relevant as the dimer calculations can provide information about both the preferred geometry and the depth of the interaction energy minimum. We therefore view this as an important direction for future work.

Reviewer #2 (Remarks on code availability):

I didn't attempt to run the code, but I can view and read the intructions. It looks fairly startighforward.

Reviewer #3 (Remarks to the Author):

This article on predicting the sold-state structures of MOCs (i.e., packing behavior) based on inexpensive computations is extremely well presented and provides a valuable contribution to the literature. The phase diagrams, in particular, are a highlight of this work. My only minor critique is that Figure 7 could benefit from the criteria for being most/least favoured being more clear in the Figure (or caption). Otherwise I recommend

publishing the paper as is.

We thank the reviewer for their kind comments and have revised the figure caption to define the colour scale more clearly: "2D schematic examples of different packing arrangements for hard particles. Green indicates that the criterion is well satisfied, yellow indicates that it is partially satisfied, and red indicates that it is poorly satisfied. For interparticle distance, "well satisfied" means close to the preferred interaction distance; for facet-to-facet contact, it means large facet overlap; and for coordination number, it means a high number of neighbouring particles." We note that this figure has now been moved to the SI based on comments from reviewer 1 (point 10) (Fig. S4).

Reviewer #4 (Remarks to the Author):

I co-reviewed this manuscript with one of the reviewers who provided the listed reports. This is part of the Nature Communications initiative to facilitate training in peer review and to provide appropriate recognition for Early Career Researchers who co-review manuscripts.
