# Supplementary material for: The role of shape and interaction directionality in the crystalline phase behaviour of octahedral metal-organic cages
Source: Nat Commun. 2026 Sep 22;17:11044. doi: 10.1038/s41467-026-77857-7 (PMC13690734; doi:10.1038/s41467-026-77857-7)
Supplement: Supplementary file 3 [file 41467_2026_77857_MOESM2_ESM.docx]

**Inventory of the Supporting Information**

| **Item** | **Description** |
| --- | --- |
| Supplementary Table 1 | Interaction parameters used in the HPMC simulations. |
| Supplementary Figure 1 | Examples of the five high-symmetry molecular-dimer configurations explored in this work. |
| Supplementary Table 2 | Relative energies of the lowest-energy dimer for each packing type across the solvent, constraint and optimisation conditions. |
| Supplementary Figure 2 | Lowest-energy constrained metal-organic-cage dimer, showing the preferred facet-to-facet packing. |
| Supplementary Figure 3 | Representative clusters of the different phases formed in the simulations. |
| Supplementary Figure 4 | Two-dimensional schematic examples of favoured packing arrangements for hard particles. |
| Supplementary Figure 5 | Phase-specific distributions of the interparticle-distance, facet-contact and coordination-number descriptors. |
| Supplementary Figure 6 | Facet-overlap distributions for neighbouring polyhedra across truncation and interaction-directionality parameters. |
| Supplementary Figure 7 | Interaction energy as a function of temperature for parameter combinations that produced multiple phases. |
| Supplementary Figure 8 | Views of the grain boundary formed in a simulation with angular width 0.7 and truncation 0. |
| Supplementary Methods | Procedure used to identify atomic overlaps and catenation in generated molecular dimers. |
| Supplementary Figure 9 | Pore-size distributions for the different structures produced in the simulations. |
